# **α**4 Integrin blockade impairs CD8^+^ T cell neuroimmune surveillance following SIV infection

**DOI:** 10.1172/JCI198730

**Published:** 2026-02-24

**Authors:** Pabitra B. Pal, Sonny R. Elizaldi, Giovanne B. Diniz, Ravi Prakash Rai, Yashavanth Shaan Lakshmanappa, Anil Verma, Daniel Rossmiller, Jesse Kaufman, Rahul Srivastava, Sean Ott, Carissa T. Erices, Kayla Schwartz, Danielle Beckman, Zhong-Min Ma, Alex Petkov, Daniel Newhouse, Dhivyaa Rajasundaram, John H. Morrison, Reben Raeman, Smita S. Iyer

**Affiliations:** 1 Department of Pathology, School of Medicine, University of Pittsburgh, Pittsburgh, Pennsylvania, USA.; 2 California National Primate Research Center, University of California, Davis, Davis, California, USA.; 3 Organ Pathobiology and Therapeutics Institute and; 4 Department of Pharmacology and Chemical Biology, School of Medicine, University of Pittsburgh, Pittsburgh, Pennsylvania, USA.; 5 Bruker Spatial Biology, Seattle, Washington, USA.; 6 Department of Pediatrics, School of Medicine, University of Pittsburgh, Pittsburgh, Pennsylvania, USA.; 7 Department of Neurology, School of Medicine, University of California, Davis, Davis, California, USA.

**Keywords:** AIDS/HIV, Immunology, Adaptive immunity

## Abstract

Integrin-targeted therapies are under investigation for HIV-associated neuroinflammation, yet their effect on CNS antiviral immunity remains undefined. We examined the role of α4 integrin in T cell–mediated neuroimmune surveillance using SIV-infected macaques with α4 blockade and T cell–specific α4-deficient mice. In macaques, α4 blockade preserved CD4^+^ Th1 cell access to the brain parenchyma but impaired CD8 effector recruitment, disrupting antiviral control. Despite stable cerebrospinal fluid viral loads, hippocampal SIV RNA increased under blockade. Single-cell analyses revealed α4 enrichment in CD8 effector memory (Tem) cells; blockade reduced inferred CD8^+^ Tem-monocyte interactions and heightened innate immune activation in the hippocampus. Microscopy demonstrated persistent SIV-induced microglial simplification despite treatment. Th1 CD4 effectors correlated positively with gray matter viral RNA, whereas α4β7^+^ CD8^+^ T cells correlated inversely, implicating impaired CD8^+^ Tem recruitment in elevated parenchymal viral burden. In mice, α4 proved dispensable for CD4 trafficking to inflamed brain but essential for CD8 effector access across CNS compartments and for both subsets to reach skull marrow. These findings establish that α4 integrin governs CD8-mediated neuroimmune surveillance through coordinated cellular positioning, with blockade enabling viral seeding while disrupting spatially organized antiviral defense.

## Abstract 2

Blocking α4 integrin impairs CD8 T cell access to the brain, weakening antiviral defense and raising caution for α4-targeted therapies in virus-driven neuroinflammation.

## Graphical abstract

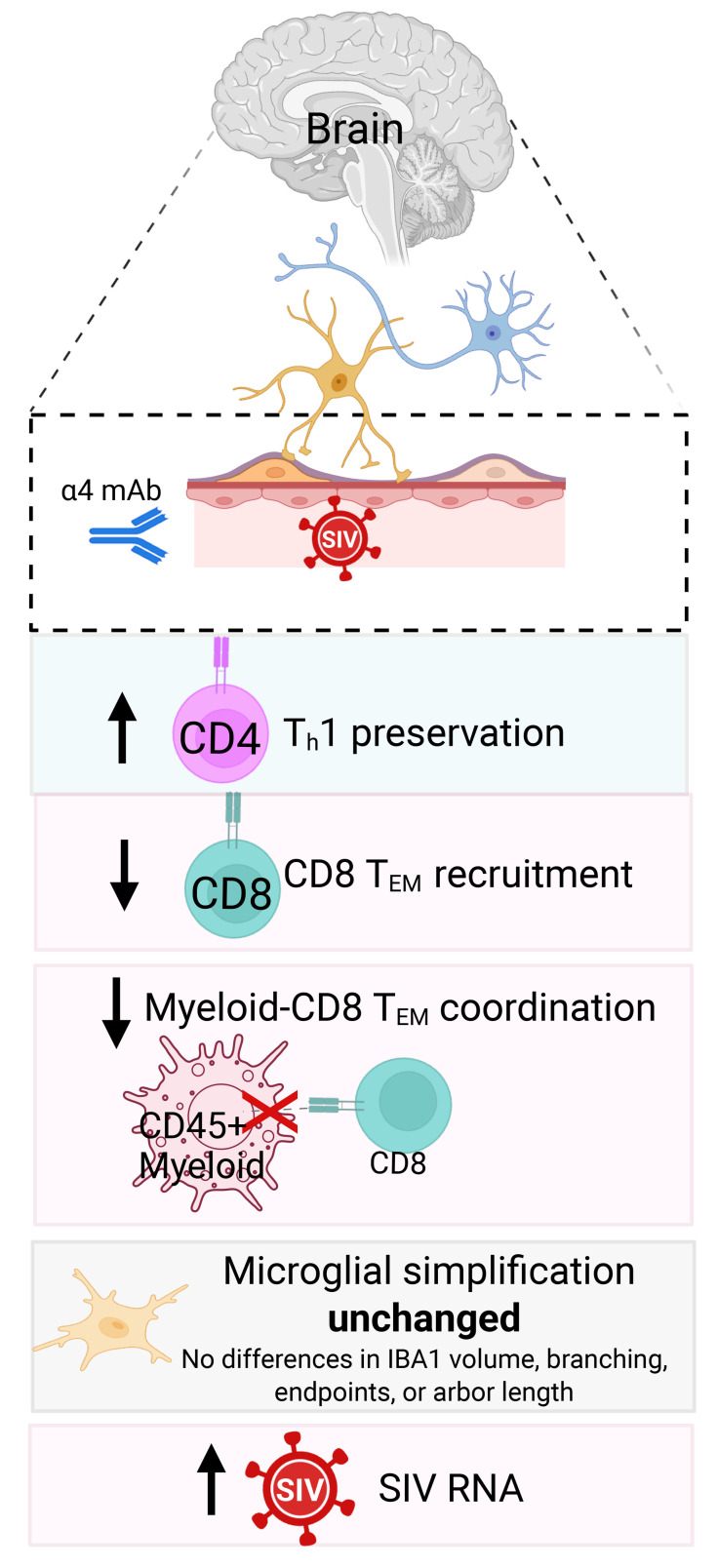

## Introduction

T cells monitor the CNS and are the predominant immune subset in the cerebrospinal fluid (CSF). They reside in brain parenchyma and drain through dural lymphatics to cervical lymph nodes (1–3). In addition to their surveillance role, T cells support CNS function by promoting neurogenesis and cognitive processes. For instance, studies have shown that CD4^+^ T cell depletion impairs neurogenesis and cognition in mice (4–6), and similarly cortical thinning in people living with HIV (PWH) correlates with low CD4 counts (7, 8), suggesting a critical role for T cells in CNS health.

However, T cells can negatively impact the CNS environment by facilitating HIV establishment and inducing neuroinflammation. In murine models, CD4^+^ T cells can establish CNS HIV infection in the absence of myeloid cells (9), through their ability to cross the blood-brain barrier and facilitate viral entry. In primates, SIV RNA is detected in the brain within days of infection, preceding viremia (10), likely via constitutive trafficking of CCR5^+^CD4^+^ Th1 cells and monocytes (9, 11–13). In PWH, CSF CD4^+^ T cells sustain acute viral replication (12), and higher levels of cell-associated viral DNA in CSF predict worse neurocognitive outcomes (14). Activated CD8^+^ T cells, enriched in the CSF during HIV infection, correlate with neuroinflammation despite being essential for antiviral control (15, 16). Thus, T cells embody a duality in the CNS. They are indispensable for maintaining homeostasis and defense. However, they also contribute to viral persistence and inflammatory injury, highlighting the intricate balance of their function.

Restricting T cell entry into the CNS may limit HIV seeding and neuroinflammation but risks weakening antiviral defense, underscoring the need for mechanistic insights to guide strategies preventing cognitive decline in PWH (17–20). CNS entry is governed by integrins and chemokine receptors, including α4β1 integrin (VLA-4), CXCR3, and CCR6, differentially expressed across effector and memory subsets. Given the link between CD4^+^ T cell α4β7 expression and HIV susceptibility (21), understanding how α4-directed therapies shape CNS immune access is essential for interpreting their effects on viral burden and immune coordination.

α4 Integrin blockade is best studied in multiple sclerosis (MS), where natalizumab reduces CNS immune cell infiltration and attenuates inflammatory lesions (22), prompting interest in applying this approach to PWH (23, 24). However, natalizumab does not block CNS entry fully, as Th1/Th17 cells can reroute through alternative P selectin glycoprotein ligand 1 (PSGL-1) or CD146 adhesion pathways (25–27), and in mouse MS models, α4 blockade sometimes exacerbates disease (28).

In macaque HIV models, α4 and α4β7 blockade increases circulating Th1 and Th17 CD4^+^ T cells, consistent with impaired homing to gut-associated lymphoid tissue (GALT), but consequences for CNS entry remain unclear (29). Prior studies report divergent effects on viral burden; combined α4 blockade and CD8^+^ T cell depletion reduces cortical viral RNA (vRNA) (30), while dual α4β1 and α4β7 antagonism increases set-point viremia (31). These discrepancies highlight the need to define how α4 integrin regulates subset-specific T cell access to the CNS and local antiviral control in an immunocompetent host.

To address this, we administered α4 blocking antibody to immunocompetent macaques 1 week before SIV infection. Blockade induced transient lymphocytosis and altered CD4^+^ T cell trafficking but had no effect on plasma or CSF viral loads. Instead, viral burden increased in gray matter (gm) regions, including the hippocampus and prefrontal cortex. Quantitative morphometry revealed that SIV infection drove structural regression of hippocampal microglia despite α4 blockade.

To define the cell-intrinsic requirement for α4 in CNS entry, we assessed CD4*^ΔItga4^* and CD8*^ΔItga4^* mice under steady state and LPS-induced inflammation. CD4^+^ T cells accessed the brain parenchyma in both settings, while CD8^+^ T cell infiltration was impaired, indicating selective CD8 effector dependence on α4. Together, our findings identify α4 integrin as a context-dependent regulator of CNS antiviral responses during SIV infection.

## Results

### α4 Blockade promotes lymphocytosis and transient Th1 CD4^+^ T cell enrichment in blood.

**Figure 1:**
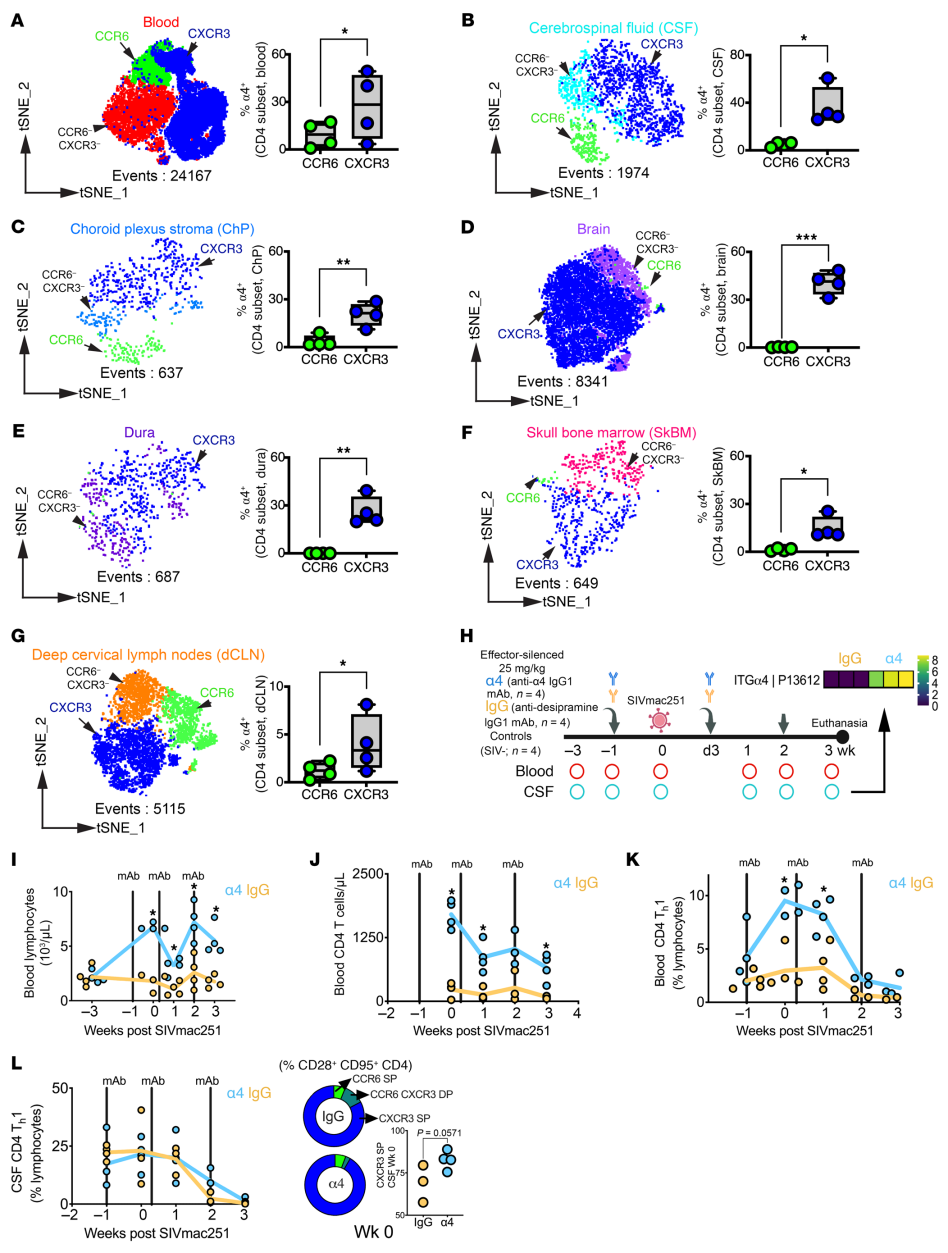
α4 Integrin expression and effects of blockade on T cells in blood and CSF. (**A**–**G**) T-distributed stochastic neighbor embedding (t-SNE) of CD95^+^α4^+^ CD4^+^ T cells across 7 tissues, color-coded by tissue. Overlays show CCR6^+^ (green; Th17) and CXCR3^+^ (blue; Th1) subsets; cells lacking CCR6 or CXCR3 retain tissue-specific coloring. Box-and-whisker plots show subset enrichment across tissues. (**H**) Experimental timeline. Rhesusized α4 mAb or IgG1 isotype control (25 mg/kg) administered at week –1 and every 10 days (arrows) to sustain α4 occupancy during viral eclipse. The inset shows *z* score–normalized mean fluorescence intensity (MFI) of infused mAb in week 3 plasma via custom autoantigen array (*n* = 3/group). SIV^–^ controls (*n* = 4) assessed at necropsy for select indices. (**I**) Total lymphocyte counts after infection. (**J**) Blood CD4^+^ T cell counts after infection. CXCR3^+^ Th1 CD4^+^ T cell frequencies in (**K**) blood and (**L**) CSF. The inset in **L** shows CXCR3^+^ and CCR6^+^CD4^+^ T cell subsets in CSF at week 0. *P < 0.05; **P < 0.01; ***P < 0.001; 1-tailed paired *t* test (**A**–**G**) or unpaired Mann-Whitney test (**L**).

We quantified α4 expression across CD4^+^ T cell subsets to identify populations licensed for CNS migration. Within the α4^+^CD95^+^ memory pool, CXCR3^+^ cells outnumbered CCR6^+^ cells, indicating preferential α4 expression on Th1 cells (Figure 1, A–G). CD28^–^ cells, typically enriched for terminal effectors, showed higher α4 expression than CD28^+^ cells in blood (median 90% vs. 40%) and skull bone marrow (94% vs. 60%) (Supplemental Figure 1A; supplemental material available online with this article; https://doi.org/10.1172/JCI198730DS1). In contrast, the CSF was enriched for CD28^+^ T cells, making α4^+^CD28^+^ subsets the most abundant among both CD4 and CD8 compartments (Supplemental Figure 1B). CXCR3^+^CD28^+^ Th1 cells constituted the dominant α4^+^ CD4 population in both blood and CSF (Supplemental Figure 1, C and D). In brain and spleen, α4 was coexpressed primarily with β1 rather than β7 (Supplemental Figure 1E). Comparison of α4β1^–^ versus α4β1^+^CD4^+^ T cells in the brain parenchyma showed higher relative expression of CCR5 and CD69 on the former, while CXCR3 and CCR6 were expressed at similar levels (Supplemental Figure 1, F and G). Together, Th1-polarized and CD28^–^ effector populations showed enriched α4 expression, licensing them for CNS or GALT migration.

To evaluate α4 integrin function in vivo, we administered 25 mg/kg anti-α4 IgG1-LALA at day −7 of SIV infection (*n* = 4). This dose achieved serum levels exceeding the threshold for complete α4 occupancy, sustaining blockade during CNS seeding. Given the half-life of natalizumab (11 ± 4 days), we administered infusions every 10 days (32). We used an IgG1 isotype control targeting desipramine (*n* = 4) and SIV^–^ controls (Figure 1H).

Weekly blood and CSF samples were collected to monitor target engagement and virologic outcomes. Plasma at week 3 after infection confirmed circulating α4 mAb in treated animals (see inset in Figure 1H). Flow cytometry confirmed anti-α4 receptor blockade on T cells in both compartments (Supplemental Figure 2A). Ex vivo incubation of preinfusion PBMCs with treated serum or CSF demonstrated α4 receptor occupancy. As expected, antibody concentrations were higher in serum than in CSF, consistent with limited IgG penetration across the blood-CSF barrier (~1,000-fold difference) (33). These findings confirm effective, sustained blockade at the time of SIV exposure.

We analyzed peripheral and CSF immune cell frequencies to assess trafficking defects. Consistent with prior reports that α4 integrin blockade impairs lymphocyte homing to GALT (29), we observed transient lymphocytosis (Figure 1I), increased circulating CD4^+^ T cells at weeks 1 and 3, and an increase in CD8^+^ T cells at week 0 (Figure 1J and Supplemental Figure 2B). Unlike α_4_β_7_ blockade, which leads to preferential accumulation of CCR6^+^CD4^+^ T cells, α4 blockade selectively increased blood Th1 CD4^+^ T cells at weeks 0 and 1, sparing CCR6^+^ CD4 and CXCR3^+^ CD8 populations (Figure 1K and Supplemental Figure 2B).

In CSF, Th1 cell frequencies remained stable, though a borderline increase in Th1/Th17 CXCR3^+^ cells at week 0 (*P* = 0.05) suggested transient reshaping of CNS-homing phenotypes (Figure 1L). By week 2, peripheral Th1 CD4^+^ T cell levels declined with progression of infection. Other immune subsets (segmented neutrophils, monocytes, eosinophils), red blood cells, and clinical parameters (hemoglobin, hematocrit, and platelet counts) remained stable between groups (Supplemental Figure 3).

### α4 Blockade rewires brain immune networks impairing viral control.

**Figure 2:**
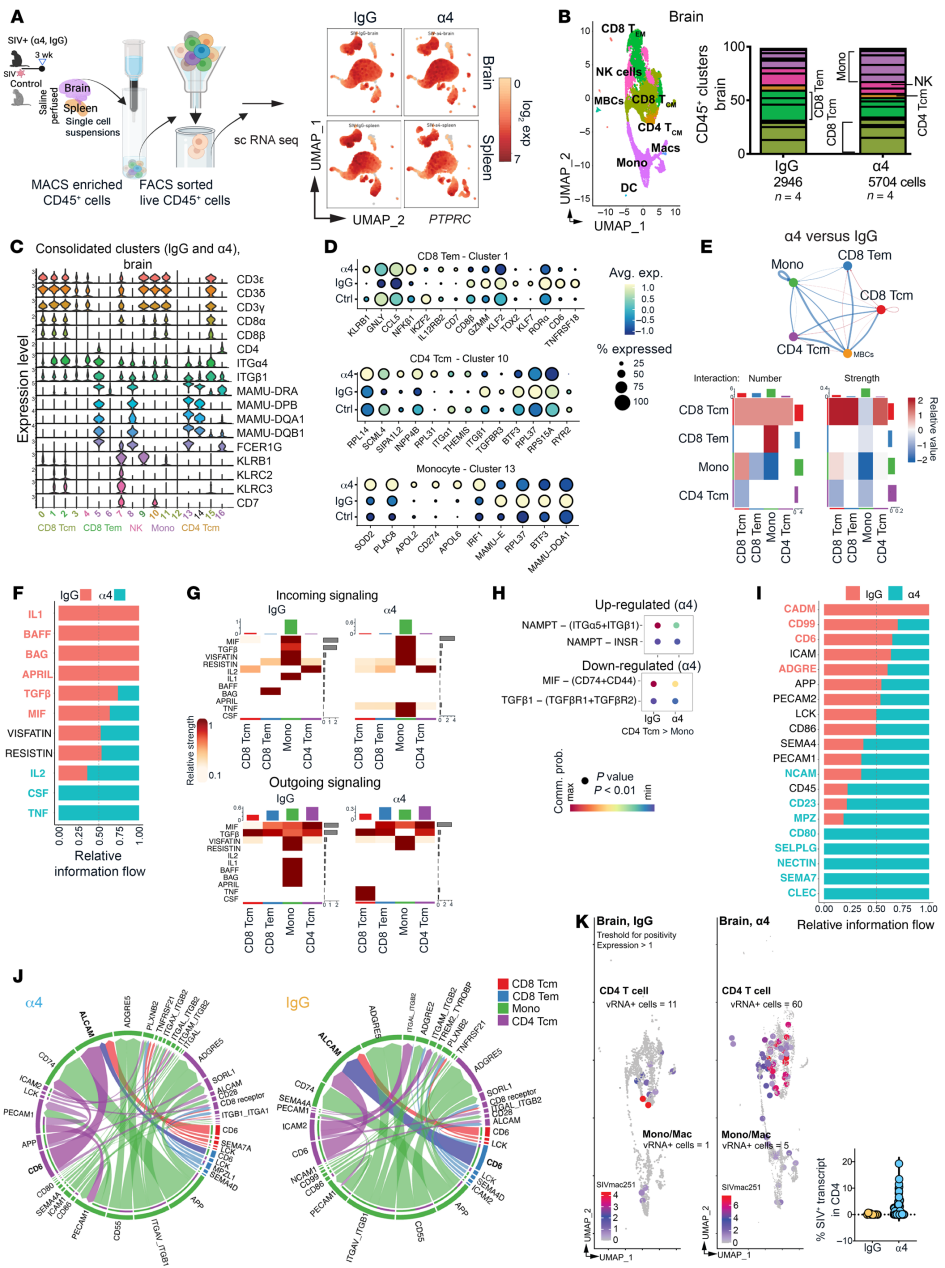
Single-cell transcriptomics reveals disrupted immune coordination and altered antiviral signaling in the brain following α4 blockade. (**A**) Single-cell RNA-seq workflow. UMAP of brain and spleen CD45^+^ cells show leukocyte enrichment by *PTPRC* expression. (**B**) Brain immune cluster annotation using Blueprint and ENCODE references identifies CD4^+^/CD8^+^ T cells, NK cells, and myeloid subsets. (**C**) *ITGA4* expression across brain immune clusters. (**D**) Dot plots comparing α4-treated, IgG-treated, and SIV^–^ control (Ctrl) groups: (top) CD8^+^ Tem gene expression, (middle) CD4^+^ Tcm gene expression, (bottom) Myeloid gene expression. (**E**) CellChat shows preserved interaction number but reduced strength for secreted signaling between CD8^+^ Tem and monocytes under α4-blockade. (**F**) Cytokine pathways enriched in IgG-treated brains (IL-1, BAFF, APRIL, BAG) versus α4-treated brains (IL-2, TNF-α, CSF), with altered CD8^+^ Tem-monocyte directionality. (**G**) Aggregate signaling shows reduced cytokine output and increased receptor input in α4-treated monocytes. (**H**) Ligand-receptor analysis shows increased NAMPT and reduced MIF-CD74/CD44 and TGFβ1-TGFβR1/TGFβR2 interactions with α4-blockade. (**I** and **J**) CD6 signaling is diminished in CD8^+^ Tem cells while LCK signaling is preserved. CD80, SELPLG, NECTIN, and SEMA7 pathways are maintained or increased. (**K**) SIV RNA^+^ cell frequencies in CD4^+^ T cell and monocyte clusters.

To define CNS-specific immune alterations following α4 blockade, we performed single-cell RNA-seq (scRNA-seq) on live CD45^+^ cells isolated from cryopreserved brain and spleen tissues, including SIV^–^ control animals (3). Cell quality metrics were consistent across conditions, with low stress-related gene expression in brain and spleen CD45^+^ cells (Supplemental Figure 4). Uniform manifold approximation and projection (UMAP) projection confirmed leukocyte enrichment based on uniform *PTPRC* (CD45) expression (Figure 2A), validating integrity of sorted cells.

Unsupervised clustering using the Blueprint and ENCODE reference identified 5 major immune populations among 20 subclusters in the brain: CD8^+^ T cells (3 effector memory [Tem], 5 central memory [Tcm] clusters), myeloid cells (6, mono), NK cells (2), and CD4^+^ T cells. Subclusters were aggregated by lineage for UMAP projection (Figure 2B). *ITGA4* was broadly expressed, highest in Tem clusters 1 and 2, monocyte cluster 5, and CD8^+^ Tcm cluster 15. Consolidated expression is shown in Figure 2C, and treatment-specific expression is shown in Supplemental Figure 5. In the spleen, 22 clusters were identified, including B cells absent from brain (Supplemental Figure 6, A–C).

We focused on CD8^+^ Tem, CD4^+^ Tcm, and monocyte clusters, populations linked to viral seeding and antiviral control. Dot plots compared differentially expressed genes between α4-blockade and IgG-treated animals, with SIV^–^ controls shown as a reference. In CD8^+^ Tem cells, *NFKB1* (2.6-fold), *GNLY* (3.6-fold), *IL12RB2* (2-fold), and *CCL5* (2.7-fold) increased with α4 treatment, indicating preserved effector priming. However, concurrent *KLRB1* upregulation (3.7-fold) and downregulation of *KLF2* (1.8-fold), *CD6* (3.0-fold), and *TNFRSF18/ICOS* (4.0-fold) suggested impaired tissue residency and costimulation (Figure 2D, top). In CD4^+^ T cells, α4 blockade increased expression of *ITGA1* (1.7-fold) and *THEMIS* (1.6-fold), genes associated with adhesion and TCR signaling. Downregulation of *ITGB1* (1.7-fold) and *TGFBR3* (1.8-fold) suggested altered cytoskeletal regulation and reduced sensitivity to TGF-β signaling. *BTF3* suppression in both brain and spleen indicated broader transcriptional disruption (Figure 2D, middle). In monocytes, α4-blockade induced *PD-L1* (*CD274*, 2.0-fold), *IRF1* (1.8-fold), and *SOD2* (2.4-fold), indicating enhanced checkpoint signaling and oxidative stress responses. *MAMU-E* (2.1-fold), *DQ-A1* (2.5-fold), and *RPL37* (2.4-fold) downregulation indicated reduced antigen presentation and translational activity (Figure 2D, bottom). Brain NK cells showed increased expression of *GNLY* (4.2-fold), *IL12RB2* (4.2-fold), and *SAMHD1* (2.4-fold) but reduced expression of *CD226* (2.4-fold), *ICOS* (3.9-fold), and *ITGA4* (1.6-fold), indicating cytotoxic readiness and reduced costimulation (Supplemental Figure 6D).

To assess how α4 blockade altered immune cell communication, we inferred ligand-receptor signaling from single-cell transcriptomes. Predicted interactions remained stable, but overall strength declined. The sharpest decline was at the monocyte-CD8^+^ Tem interface (Figure 2E and Supplemental Figure 7A). Cytokine signaling showed higher IL-1, BAFF, APRIL, and BAG activity in IgG-treated animals, reflecting greater monocyte cytokine output (Figure 2, F and G). With α4-blockade, IL-2, TNF-α, and CSF signaling increased, driven by increased IL-2 input to CD8^+^ Tem cells, TNF-α input to CD4/CD8 clusters and monocytes, and CSF signaling from CD8^+^ Tcm cells to monocytes. Ligand-receptor analysis showed selective CD4^+^ Tcm-monocyte remodeling, with increased NAMPT but reduced MIF-CD74^+^CD44 and TGFβ1-TGFβR1^+^TGFβR2 signaling, indicating diminished monocyte immune regulation and tissue repair (Figure 2H).

Contact-mediated signaling revealed numerically higher predicted interactions following α4 blockade (134 [a4] vs. 90 [1gG]), with similar overall strength (Supplemental Figure 7B). CD6 signaling decreased in CD8^+^ Tem cells, suggesting impaired synapse formation. LCK signaling remained intact across T cell subsets, reflecting preserved proximal TCR activation (Figure 2I). CD80, SELPLG, NECTIN, and SEMA7 pathways were maintained or increased, suggesting compensatory adhesion and costimulation. Chord plots showed a shift in network topology: ALCAM-CD6 interactions between myeloid cells and CD4^+^ Tcm cells replaced CD8^+^ Tem-monocyte interactions seen with IgG treatment (Figure 2J). This shift coincided with higher SIV RNA^+^ cell frequencies in CD4^+^ T cell and monocyte clusters in α4-treated brains (Figure 2K), linking disrupted CD8^+^ Tem-monocyte connectivity to impaired viral control.

### α4 Blockade amplifies neuroimmune activation in SIV^+^ hippocampal brain regions.

**Figure 3:**
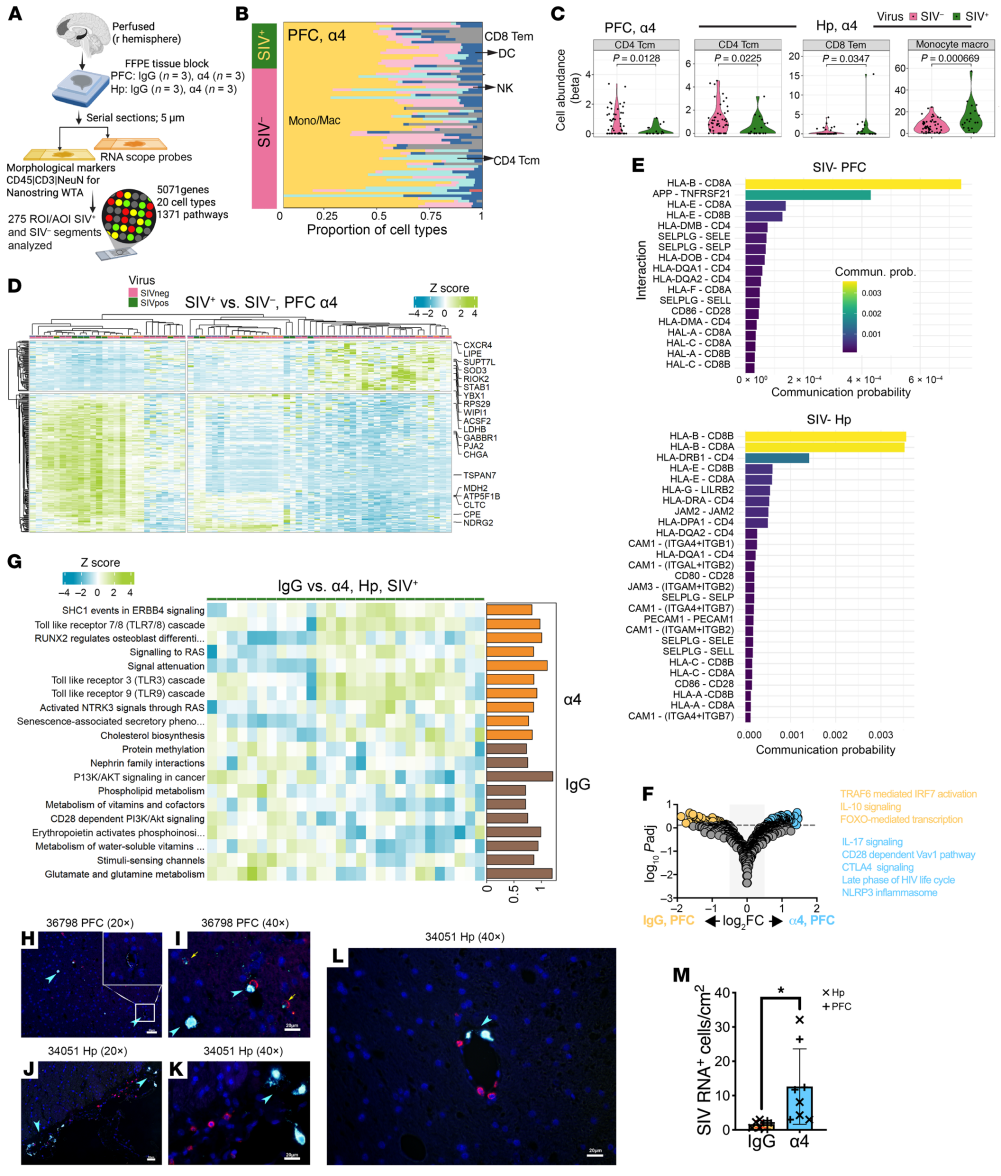
Neuroimmune activation and spatial immune organization in prefrontal cortex and hippocampus following α4 blockade. (**A**) Spatial transcriptomic workflow. RNAscope identified SIV RNA^+^ regions in prefrontal cortex (PFC) and hippocampus (Hp), guiding region of interest (ROI) selection for NanoString whole-transcriptome analysis (WTA). (**B**) Representative spatial deconvolution of SIV^+^ PFC ROI using a scRNA-seq–derived CD45^+^ brain reference. (**C**) Violin plots of immune subsets in SIV^+^ ROIs. α4-treated animals show reduced Tcm cells in PFC and Hp, with increased CD8^+^ Tem cells and monocytes in Hp versus IgG-treated animals. (**D**) Heatmap of differentially expressed genes across SIV^+^ and SIV^–^ PFC ROIs from α4-treated animals (*P* < 0.05, |log_2_FC| > 0.5). (**E**) CellChat ligand-receptor interactions in SIV^–^ PFC and Hp ROIs from α4-treated animals. (**F**) Volcano plot of pathway differences in PFC comparing α4-treated and IgG-treated groups. (**G**) Heatmap of top upregulated pathways in SIV^+^ Hp ROIs comparing α4-treated and IgG-treated animals. (**H**–**L**) RNAscope detection of SIV RNA in brain parenchyma and perivascular regions. Representative PFC images show SIV RNA^+^ signal (cyan) and CD3^+^ T cells (red). Yellow arrow indicates CD3^+^ SIV RNA^+^ cell; cyan arrowheads indicate SIV RNA^+^ cells near perivascular spaces. Magnified views of **H** and **J** on the left are shown in **I** and **K**. Scale bars: 50 μm (**H**, **J**, and **L**); 20 μm (**I** and **K**). (**M**) SIV RNA^+^ cells per cm² in PFC and Hp (mean ± SD). *P < 0.05, 2-tailed Welch’s *t* test.

To investigate regional effects of α4 blockade on brain inflammation, we performed spatial transcriptomic profiling of the prefrontal cortex (PFC) and hippocampus (Hp) from α4- and IgG-treated macaques (*n* = 3/group). RNAscope-guided selection of SIV RNA^+^ areas informed sampling of 275 regions of interest (ROIs) for Nanostring Whole Transcriptome Atlas analysis (Figure 3A and Supplemental Figure 8). Dimensionality reduction showed that SIV RNA status, not treatment, drove most variance (Supplemental Figure 9, A and B).

Spatial deconvolution using the CD45^+^ brain scRNA-seq reference (3) showed comparable marker detection across groups within SIV^–^ and SIV^+^ regions, validating immune cell representation (Supplemental Figure 9C). We focused on immune subsets relevant to viral entry and control from the scRNA-seq analysis: CD4^+^ Tcm, CD8^+^ Tem, and monocyte cells. These subsets were detected in both SIV^–^ and SIV^+^ regions across groups (Figure 3B and Supplemental Figure 9D). Within α4-treated animals, SIV^+^ regions showed reduced CD4^+^ Tcm cells in both PFC and Hp, with increased CD8^+^ Tem cells and monocytes in SIV^+^ Hp (Figure 3C), patterns absent in IgG-treated animals (data not shown)

Differential expression analysis between SIV^+^ and SIV^–^ PFC ROIs identified *n* = 269 significantly differentially expressed genes in α4-treated animals (*P* < 0.05, log_2_ fold change [FC] > 0.5) (Figure 3D and Supplemental Figure 9E). SIV^+^ regions upregulated genes for neuronal structure (*MAP2*), synaptic activity (*SNAP25*), metabolism (*COX6C*), and antiviral responses (*SAMHD1, IRF1*), alongside IL-17, TLR, and inflammasome signaling. SIV^–^ regions were enriched for transcriptional and oxidative stress regulators. In Hp, 311 differentially expressed genes were enriched for neuronal, synaptic, and stress response pathways (Supplemental Figure 9F).

Within SIV^–^ regions, CellChat predicted reduced monocyte-CD8^+^ Tem interactions following α4 blockade, with enriched alternative adhesion pathways. Ligand-receptor analysis of SIV^–^ regions confirmed preserved antigen recognition and compensatory adhesion in α4-treated animals (Figure 3E), though these were insufficient to restore antiviral immunity in SIV^+^ regions.

We next examined transcriptional responses in SIV^+^ ROIs, beginning with the PFC. Between-group comparison revealed distinct PFC signatures: α4 blockade induced HIV late-phase, IL-17, and NLRP3-associated genes, while IgG treatment enhanced IRF7 and IL-10 signaling (Figure 3F). In SIV^+^ Hp, α4 blockade induced TLR7/8 transcripts, whereas IgG treatment preserved CD28 signaling (Figure 3G). In situ hybridization confirmed SIV^+^CD3^+^ T cells in perivascular and parenchymal compartments (Figure 3, H–L), with increased SIV RNA signal in α4-blockade regions (Figure 3M). Together, α4 blockade shifted neuroimmune responses from coordinated adaptive immunity toward innate immune activation, particularly in Hp, creating conditions permissive for viral persistence.

### α4 Blockade elevates viral load in cortical gm regions.

**Figure 4:**
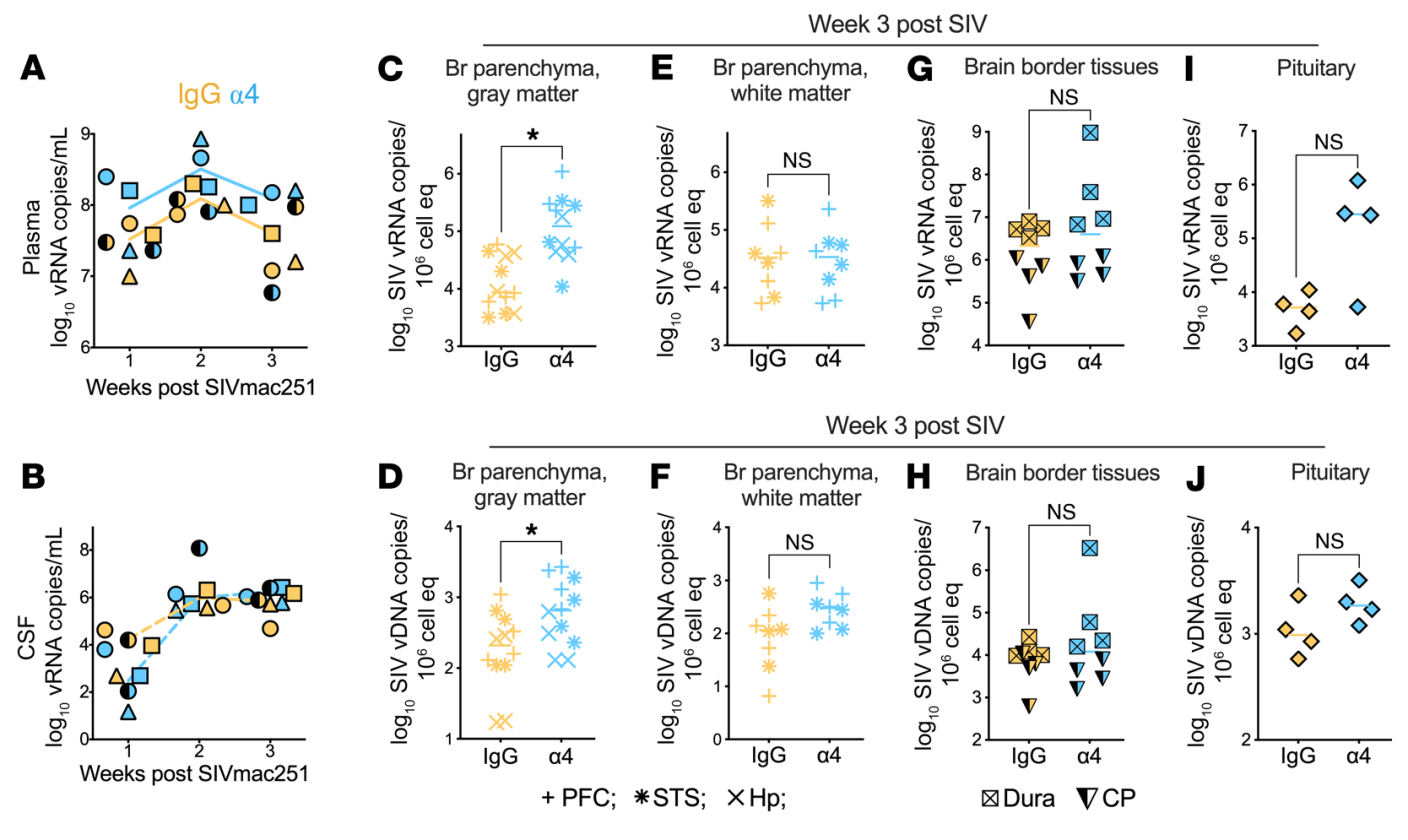
α4 Integrin blockade increases viral burden in brain parenchymal gray matter. (**A**) Plasma and (**B)** cerebrospinal fluid (CSF) viral RNA levels over time. (**C**) SIV RNA and (**D**) SIV DNA levels in gray matter regions, including hippocampus (Hp) and superior temporal sulcus (STS), demonstrating increased viral burden in α4-treated animals. (**E**) SIV RNA and (**F**) SIV DNA levels in neocortical white matter showing no significant differences between groups. (**G** and **I**) SIV RNA and (**H** and **J**) SIV DNA levels in border-associated tissues, including pituitary and dura. *P < 0.05 by unpaired *t* test.

To assess the effect of α4 blockade on viral kinetics, we quantified plasma and CSF vRNA longitudinally. Peak viremia at week 2 was similar between α4- and IgG-treated animals (plasma: α4, 1.1 × 10^8^ vs. IgG, 3.2 × 10^8^; CSF: α4, 4.7 × 10^5^ vs. IgG, 9.7 × 10^5^), with similar viral trajectories (Figure 4, A and B, and Supplemental Figure 10). One α4-treated macaque (ID 41217) showed transient suppression of CSF vRNA at week 1, but CNS entry was consistently achieved across treatment groups.

We next examined whether α4 blockade increased tissue viral burden, as predicted by the scRNA-seq and spatial transcriptomic data. SIV RNA and DNA were significantly increased in gm regions, including the Hp and superior temporal sulcus (*P* < 0.05; Figure 4, C and D), implicating enhanced parenchymal seeding or reduced clearance. In contrast, vRNA in neocortical white matter (wm), dura, and pituitary were comparable across groups (Figure 4, E–J), with modest but nonsignificant pituitary vDNA increase with blockade. These data show that α4 blockade does not restrict CNS entry of SIV^+^ cells and instead permits viral accumulation in select gm regions where immune containment may be disrupted.

### α4 Blockade does not attenuate SIV-induced microglial activation in hippocampal gm.

**Figure 5:**
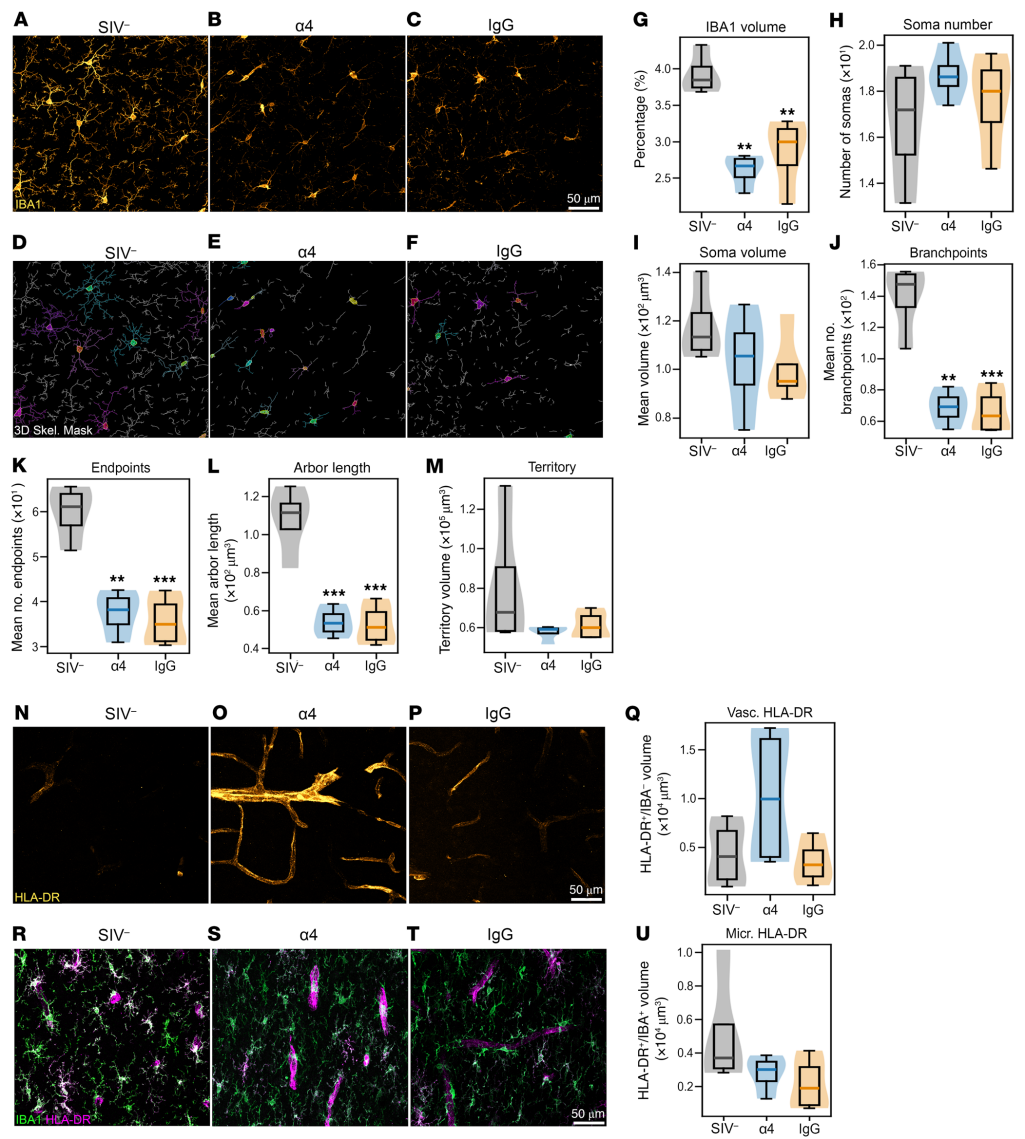
α4 Blockade does not attenuate SIV-induced microglial activation in hippocampal gray matter. (**A**–**C**) Hippocampal CA1 IBA1 immunolabeling. SIV^–^ controls show ramified microglia with organized territorial distribution. SIV-infected animals (α4- and IgG-treated) show reduced arborization, fewer branches, and irregular soma morphology, indicating persistent activation; α4 blockade did not restore resting morphology. (**D**–**F**) Maximum intensity projection of 3D segmentation masks for morphometric analysis. (**G**–**M**) Microglial morphology quantification in hippocampal formation (*n* = 4/group; mean of 24 fields/animal). Infected animals show reduced complexity: decreased relative IBA1 volume without reduced microglial number, fewer branch points and endpoints, and reduced arbor length. (**N**–**P**) PFC gray matter HLA-DR immunolabeling. α4-Treated animals show increased HLA-DR signal with vessel-associated morphology versus SIV^–^ controls. (**Q**) HLA-DR quantification in PFC gray matter shows no significant differences due to high intra-animal variability (*n* = 4/group; mean of 12 fields/animal). (**R**–**T**) PFC white matter IBA1/HLA-DR coimmunolabeling. In SIV^–^ animals, HLA-DR colocalizes with IBA1^+^ microglia; in α4- and IgG-treated animals, HLA-DR associates more with IBA1^–^ vessel-shaped structures. (**U**) HLA-DR/IBA1 colocalization quantification shows no significant differences between treatments (*n* = 4/group; mean of 12 fields/animal). **P < 0.01; ***P < 0.001 by 1-way ANOVA with Tukey’s HSD. Scale bar: 50 μm.

To determine whether microglial activation was affected with blockade, we examined the hippocampal formation (HF) and PFC gm and wm matter. Quantitative morphometry revealed a significant SIV effect on IBA1 volume in the HF (ANOVA *P* = 0.0006), but no difference between α4 and IgG-treated animals (*P* = 0.48). These effects remained significant when normalized to slice volume (Figure 5, A–M**)**. Despite reduced IBA1 volume, SIV infection did not impact microglial somas number or size in the HF (Figure 5, H and I). Multiple morphometric indices supported microglial simplification in HF. SIV infection significantly decreased microglial branchpoints (ANOVA: *P* = 0.0004), with reduced branching complexity in both α4 and IgG animals versus controls (IgG vs. SIV^–^, *P* = 0.0008; α4 vs. SIV^–^, *P* = 0.001) (Figure 5J). Similarly, microglial endpoints (ANOVA: *P* = 0.0005) and average arbor length (ANOVA: *P* = 0.0003) were significantly reduced. Tukey’s post hoc comparisons confirmed that α4- and IgG-treated animals (Figure 5, K and L) had significantly shorter arbors than controls (IgG vs. SIV^–^, *P* = 0.0007; α4 vs. SIV^–^, *P* = 0.0008). Thus, pronounced simplification of microglial morphology and process retraction ensued after SIV, consistent with activation (34, 35). The absence of significant infection effects on soma or territory volume (Kruskal-Wallis: H = 1.65, *P* = 0.48) suggests that microglia did not fully transition to an ameboid morphology associated with phagocytic activation (Figure 5M). Importantly, microglia in α4- and IgG-treated animals were morphologically indistinguishable, indicating limited effects of α4 blockade. No significant changes in microglial spatial distribution were observed across multiple metrics (Clark-Evans R: *P* = 0.066; Clark-Evans Z: *P* = 0.28; Hopkins H: *P* = 0.24; L–r AUC: *P* = 0.76).

HLA-DR immunolabeling revealed high interanimal and intersectional variability, consistent with focal rather than widespread expression changes. SIV infection did not significantly alter HLA-DR metrics in the HF (HLA-DR total volume: *P* = 0.44; number of HLA-DR^+^ objects: *P* = 0.54; HLA-DR object size: *P* = 0.36; HLA-DR^+^/IBA1^–^ volume: *P* = 0.44; HLADR^+^/IBA1^+^ volume: *P* = 0.55). Across animals, HLA-DR expression increased in PFC gm blood vessels (Figure 5, N–Q), but no significant SIV effect was detected on mean HLA-DR^+^/IBA1^–^ volume (ANOVA: *P* = 0.24). Similarly, no group differences were observed in HF (ANOVA: *P* = 0.44) or PFC wm (ANOVA: *P* = 0.41). A trend toward reduced microglial HLA-DR in PFC wm did not reach statistical significance (ANOVA: *P* = 0.16). Colocalization analyses showed no effect of blockade on HLA-DR^+^/IBA1^+^ volume in HF (ANOVA: *P* = 0.55) or PFC gm (Kruskal-Wallis: *P* = 0.36). Thus, SIV-associated alterations in HLA-DR were spatially heterogeneous in the PFC wm, where localized downregulation was not corrected by blockade (Figure 5, R–U).

### α4 Blockade reduces α4β1^+^ CD4 entry while preserving Th1 access.

**Figure 6:**
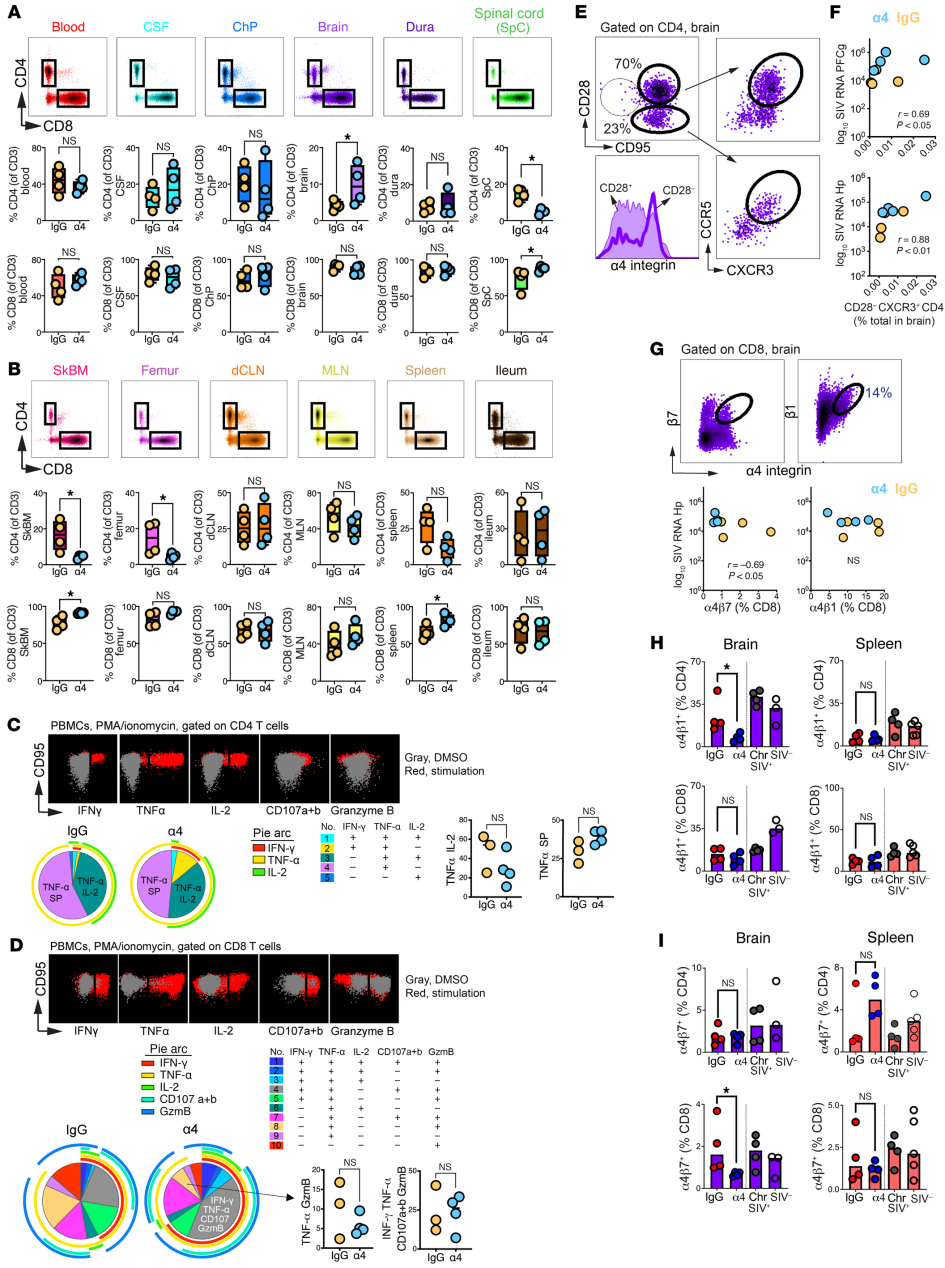
α4 Blockade selectively reduces integrin-dependent T cell recruitment to the brain parenchyma. (**A**) Frequencies of total CD4^+^ T cells in brain parenchyma of α4- and IgG-treated macaques at week 3 after infection. (**B**) CD4^+^ T cell frequencies in spinal cord, skull marrow, and femur. Total PBMCs were stimulated with phorbol 12-myristate 13-acetate (PMA) plus ionomycin for 5 hours. Representative intracellular staining profiles shown for cytokine and cytolytic molecules in (**C**) CD4^+^ T cells and (**D**) CD8^+^ T cells (*n* = 3, IgG; *n* = 4, α4). (**E**) Flow plot of CCR5/CXCR3 expression in CD28^–^ and CD28^+^ CD4^+^ T cells in brain. (**F**) Correlation between CD28^–^CXCR3^+^CD4^+^ T cell frequency and vRNA copies in hippocampal and PFC gray matter (Spearman). (**G**) Inverse correlation between α4β7^+^CD8^+^ T cell frequency and hippocampal vRNA (Spearman). (**H** and **I**) Frequencies of (**I**) α4β7^+^ and (**H**) α4β1^+^ CD4^+^ and CD8^+^ T cells in brain parenchyma and spleen Chr, chronic SIV. Data represent individual animals; lines indicate medians. *P < 0.05, unpaired *t* test. Experiments are representative of at least 3 independent replicates.

To determine whether α4 blockade altered local immune surveillance, we profiled parenchymal immune composition at week 3 after infection. Myeloid cell frequencies, including microglia and macrophages, were unchanged between groups (Supplemental Figure 11, A and B), corroborating microscopy findings that innate surveillance remained intact. In contrast, α4 blockade mitigated SIV-associated CD4^+^ T cell depletion in the brain parenchyma (Figure 6A) but not in the spinal cord, skull marrow, or femur (Figure 6B). A compensatory increase in CD8^+^ T cells in spinal cord and skull marrow was observed, indicating compartment-specific dependence on α4 integrin for T cell access.

To assess whether cytokine production in T cells was decreased following blockade, we measured T cell responses to Gag, Env, and Pol in blood, as sufficient cells for stimulation were unavailable from the brain. Conventional ICS detected no Env-specific responses, and Pol or Gag responses were observed in only 1 animal (37164, IgG) and were restricted to CD8^+^ T cells (Supplemental Figure 11C). This pattern is consistent with antigen-specific T cell responses, typically peaking around week 8 after infection (36, 37).

To determine whether the absence of detectable antigen-specific responses reflected impaired cytokine production capacity rather than limited generation of antigen-specific T cells, we assessed cytokine production together with CD107a/b and granzyme B expression following PMA and ionomycin stimulation. CD4^+^ and CD8^+^ T cells in both groups exhibited preserved cytokine and effector responses to PMA and ionomycin. In CD4^+^ T cells, responses were dominated by TNF-α and IL-2, with minimal IFN-γ and limited polyfunctionality (Figure 6C). CD8^+^ T cells showed high granzyme B expression, largely independent of stimulation, together with readily detectable CD107a/b, IFN-γ, and TNF-α responses, indicating intact cytotoxic effector potential during α4 blockade (Figure 6D).

Within the brain, blockade mitigated Th1 depletion, increasing CXCR3^+^CD4^+^ T cell frequencies while maintaining stable CD28 and CCR5/CCR7 subset proportions (Supplemental Figure 11, D and E). Because CCR5^+^CD4^+^ T cells concentrate within the Th1 pool (Figure 6E), we reasoned that their enrichment could sustain viral replication. Given elevated α4 expression on CD28^–^ effectors, we examined CD28-defined subsets and found that CD28^–^CXCR3^+^CD4^+^ T cell frequencies, but not CD28^+^ cells, correlated positively with vRNA in hippocampal and PFC gm, though not in PFC wm (Figure 6F).

To assess associations between CD8^+^ T cells and viral load, we examined integrin-defined subsets. In the Hp, α4β7^+^ but not α4β1^+^ CD8^+^ T cell frequencies correlated inversely with vRNA, implicating α4β7-mediated trafficking in local cytotoxic containment (Figure 6G). These correlations, however, being based on small group sizes should be interpreted with caution. α4 Blockade significantly reduced α4β1^+^CD4^+^ T cells in the brain parenchyma (Figure 6H), while α4β7^+^CD8^+^ T cells were similarly diminished, indicating selective impairment of integrin-dependent recruitment (Figure 6I).

In summary, α4 blockade preserved high Th1 frequencies in the brain rather than restricting CD4^+^ T cell access, suggesting early viral seeding proceeded unimpeded. The concomitant increase in parenchymal viral burden, coupled with loss of α4β7^+^CD8^+^ T cells, implicates α4 integrin in recruiting CD8 effectors to the CNS. To directly test this requirement, we employed a conditional knockout mouse model with α4 selectively deleted in CD4^+^ or CD8^+^ T cells, using LPS-induced neuroinflammation to define lineage-specific α4 dependence for CNS trafficking.

### α4 Integrin permits CD4^+^ T cell access to CNS borders but is dispensable for parenchymal entry.

**Figure 7:**
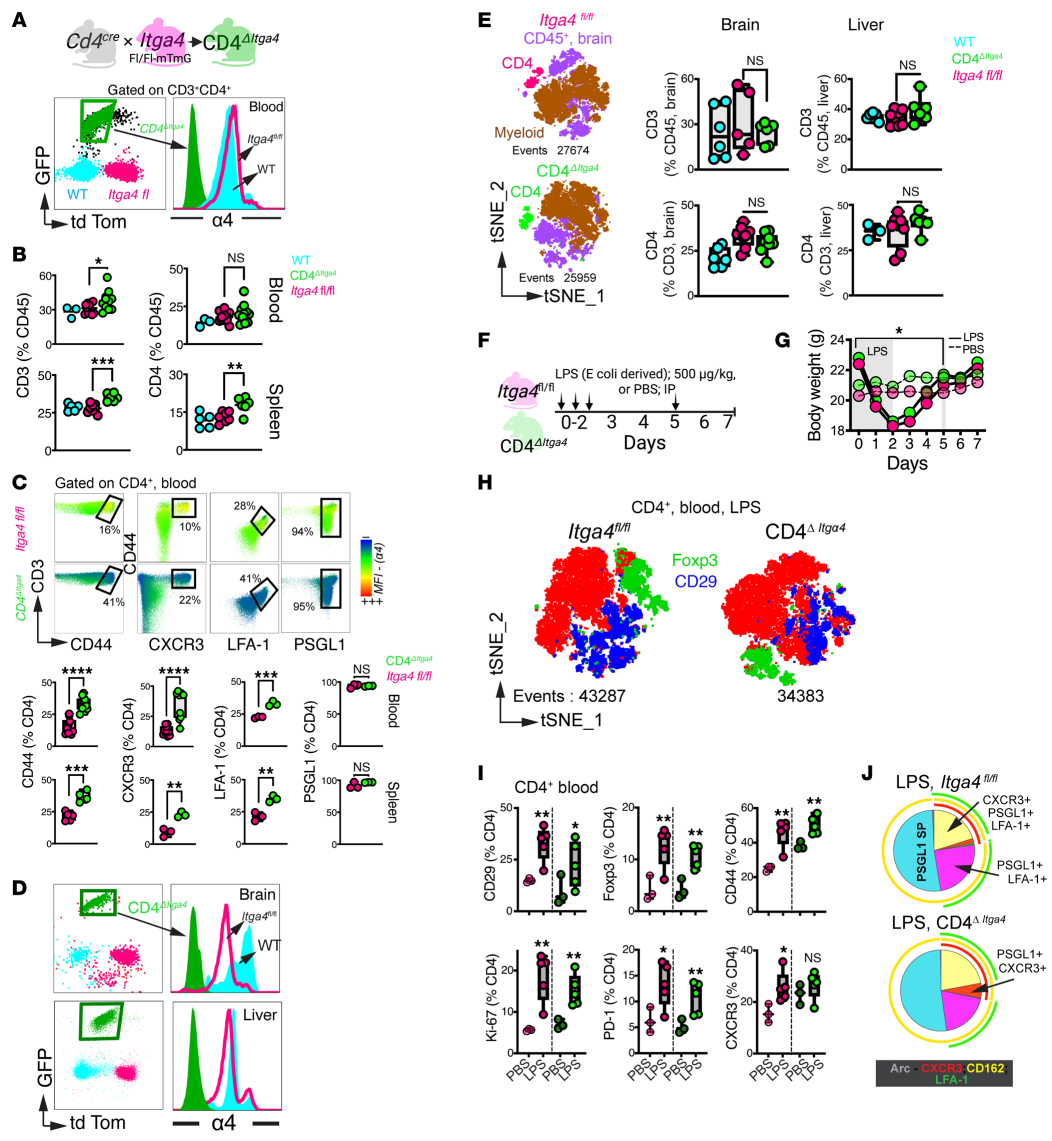
α4 Integrin is dispensable for CD4^+^ T cell access to the brain parenchyma at homeostasis. (**A**) CD4^ΔItga4^ dual-reporter system: Cre-mediated *Itga4* deletion induces tdTomato-to-GFP switch. Flow plots identify GFP^+^tdTomato^–^ CD4^+^ T cells lacking surface α4. (**B**) CD4^+^ T cell frequencies in blood and spleen at steady state. (**C**) Phenotypic profiling of circulating and splenic CD4^+^ T cells; color intensity reflects α4 MFI. (**D**) Flow plots and quantification of GFP^+^CD4^+^ T cells in brain parenchyma and liver at steady state. (**E**) t-SNE of CD45^+^ brain cells identifying CD4^+^ T cell populations by reporter status (tdTomato^+^, pink; GFP^+^, green) and myeloid cells (brown). Scatterplots show CD3 and CD4 frequencies in brain and liver. (**F**) Experimental design for LPS-induced systemic inflammation (i.p.). (**G**) Body weight loss from days 1–5 after LPS (solid line, LPS; dashed line, PBS). (**H**) t-SNE of blood CD4^+^ T cells after LPS showing genotypes, highlighting clustering of CD29^+^ (blue) and Foxp3^+^ (green) populations. (**I**) Phenotypic profiling of blood CD4^+^ T cells after LPS showing comparable induction of activation and trafficking markers across genotypes. (**J**) Boolean analysis of CXCR3, LFA-1, and PSGL-1 in blood CD4^+^ T cells showing preserved CNS-trafficking profiles. *n* = 3–8 animals per group from ≥3 independent experiments. **P < 0.01; ***P < 0.001, 1-tailed unpaired *t* test (**B**); *P < 0.05, paired *t* test (**G**) *P < 0.05, **P < 0.01; ***P < 0.001; ****P < 0.0001. One-tailed, unpaired *t*-test was used in **C** and **I**. Experiments are representative of at least 3 independent replicates.

To test the requirement for α4 in CNS T cell entry, we generated CD4*^Cre^Itga4*^fl/fl:mT/mG^ (CD4*^ΔItga4^*) and CD8*^Cre^Itga4*^fl/fl:mT/mG^ (CD8*^ΔItga4^*) mice, dual-reporter strains enabling lineage tracing via tdTomato-to-GFP switch upon α4 deletion. GFP^+^tdTomato^–^CD4^+^ T cells lacked α4, confirming efficient deletion (Figure 7A). CD4^ΔItga4^ T cell development was intact, consistent with prior reports in global *Itga4*^–/–^ mice (38, 39). CD4^ΔItga4^ T cells accumulated in blood and spleen and were reduced in Peyer’s patches (Figure 7B and Supplemental Figure 12A), mirroring redistribution observed with α4 blockade in macaques. Blood CD4^ΔItga4^ T cells showed increased CD44, CXCR3, and LFA-1 expression (Figure 7C), consistent with compensatory adhesion and chemotactic programs (40, 41). PSGL-1, PD-1, Foxp3, cytokine production, and memory subsets were unchanged (Supplemental Figure 12, B and C). CD4^ΔItga4^ T cell frequencies in brain and liver were preserved (Figure 7, D and E), and brain myeloid composition was unchanged (Supplemental Figure 12D), indicating that α4 is dispensable for constitutive CD4^+^ T cell entry into the brain parenchyma.

**Figure 8:**
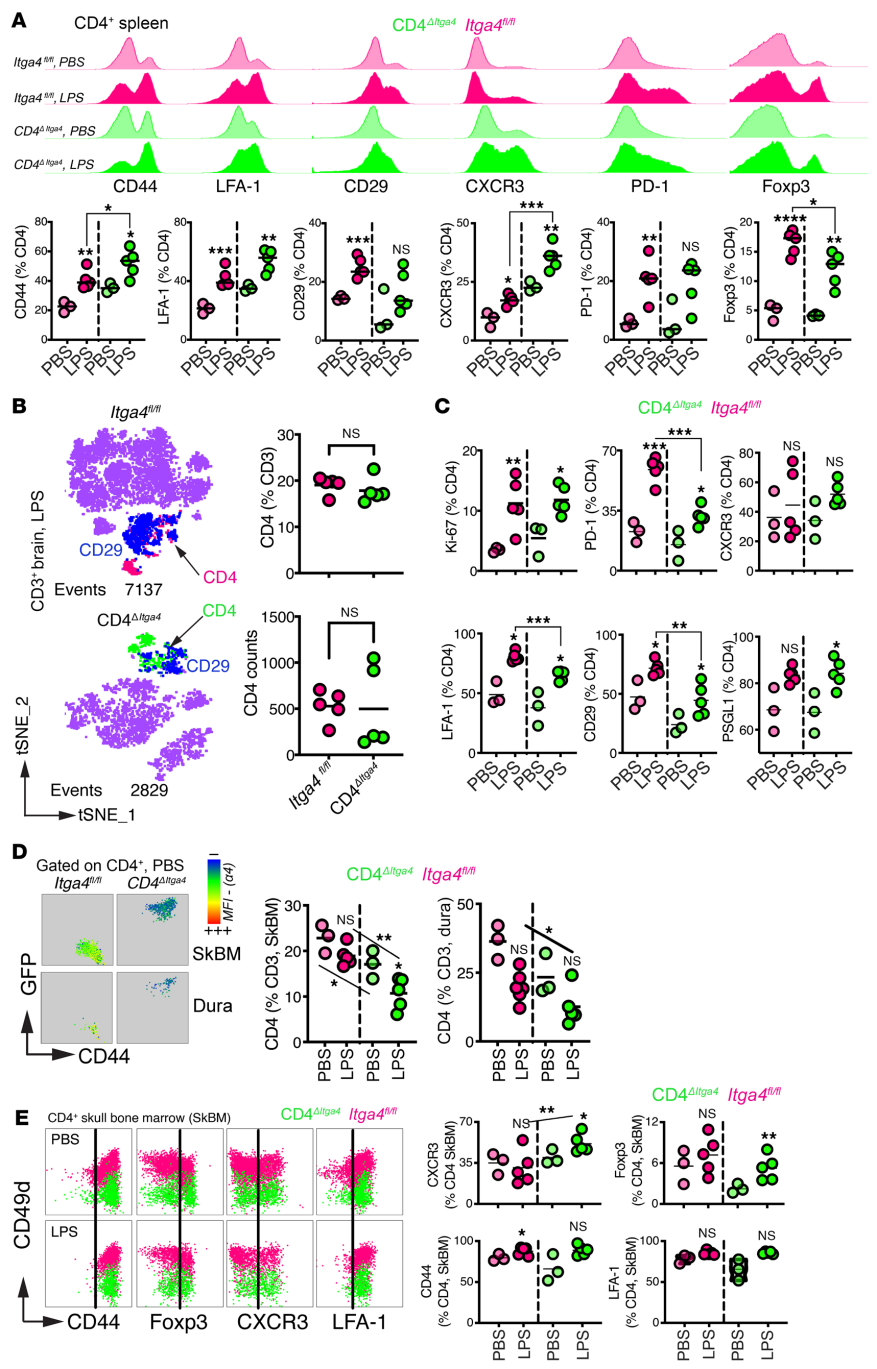
α4 Integrin is dispensable for CD4^+^ T cell access to the brain parenchyma during systemic inflammation. (**A**) Activation and trafficking marker expression in splenic CD4^+^ T cells after LPS. Histograms: GFP^+^ (green) and tdTomato^+^ (pink) CD4^+^ T cells; scatterplots quantify marker frequencies. (**B**) t-SNE of CD3^+^ brain cells showing CD4^+^ T cells by reporter status (tdTomato^+^, pink; GFP^+^, green) with CD29 overlay (blue). Scatterplots show CD4 frequencies and counts in brain. (**C**) Phenotypic profiling of brain-infiltrating CD4^+^ T cells after LPS across genotypes. (**D**) Heatmap showing α4 MFI across CD4^+^ T cell populations in skull marrow and dura. Scatterplots show CD4 frequencies in PBS- and LPS-treated mice. (**E**) Flow plots and quantification of CD4^+^ T cell phenotype in skull marrow (GFP^+^, green; tdTomato^+^, pink). *n* = 3–5 animals per group from ≥3 independent experiments. *P < 0.05; **P < 0.01; ***P < 0.001; ****P < 0.0001, unpaired *t* test unless otherwise indicated. Experiments are representative of at least 3 independent replicates.

To isolate α4-dependent trafficking mechanisms during neuroinflammation while avoiding confounding CD4 depletion in HIV infection models, we used LPS-induced neuroinflammation, which recapitulates systemic and CNS immune activation seen in acute HIV (42–44) (Figure 7F). CD4^ΔItga4^ and *Itga4*^fl/fl^ mice exhibited comparable weight loss (Figure 7G). Blood T cell activation and induction of β1 (CD29), Foxp3, and effector markers (CD44, Ki-67, PD-1, CXCR3) (Figure 7, H and I) were similar across genotypes, indicating that α4 is dispensable for CD4^+^ T cell effector differentiation during systemic inflammation. To test whether α4 contributes to acquisition of a CNS-trafficking phenotype, we performed Boolean analysis of CXCR3, LFA-1, and PSGL-1. Most circulating CD4^+^ T cells expressed PSGL-1, with subsets coexpressing LFA-1 and/or CXCR3. These profiles were preserved or modestly enriched in CD4^ΔItga4^ T cells (Figure 7J), indicating that activation and migratory programing proceed independently of α4 signaling. In the spleen, LPS induced CD44, LFA-1, CD29, CXCR3, PD-1, and Foxp3 expression in both genotypes; however, CD44 and CXCR3 frequencies were higher in CD4^ΔItga4^ mice, whereas Foxp3 frequencies were lower, indicating partial α4 dependence in helper T cell differentiation (Figure 8A).

Within the brain, CD4^ΔItga4^ T cells infiltrated the brain parenchyma comparably to α4^fl/fl^ controls (Figure 8B). Ki-67 and CXCR3 expression confirmed CD4^ΔItga4^ effector access to inflamed parenchyma (Figure 8, B and C). Although CD29 (β1) and LFA-1 were reduced, PSGL-1 was upregulated, suggesting compensatory selectin-mediated adhesion. This aligned with CellChat-inferred selectin pathway enrichment in macaque data α4 blockade (Figure 2I and Figure 3C), supporting cross-species concordance.

In contrast, CD4^ΔItga4^ T cell frequencies were reduced in CNS border compartments, skull marrow, and dura, where α4 expression was high (Figure 8D and Supplemental Figure 12E), mirroring macaque α4-blockade findings and indicating compartment-specific α4 requirements. Residual skull marrow CD4^+^ T cells retained a Th1-like phenotype with CXCR3 expression (Figure 8E).

Liver recruitment was unaffected (Supplemental Figure 12F), indicating that α4 selectively regulates CNS border access rather than general tissue homing. Together, these data show that α4 integrin is dispensable for CD4^+^ T cell infiltration of brain parenchyma during inflammation but required for skull marrow access.

### CD8^+^ T cell effectors require α4 integrin for CNS entry during inflammation.

**Figure 9:**
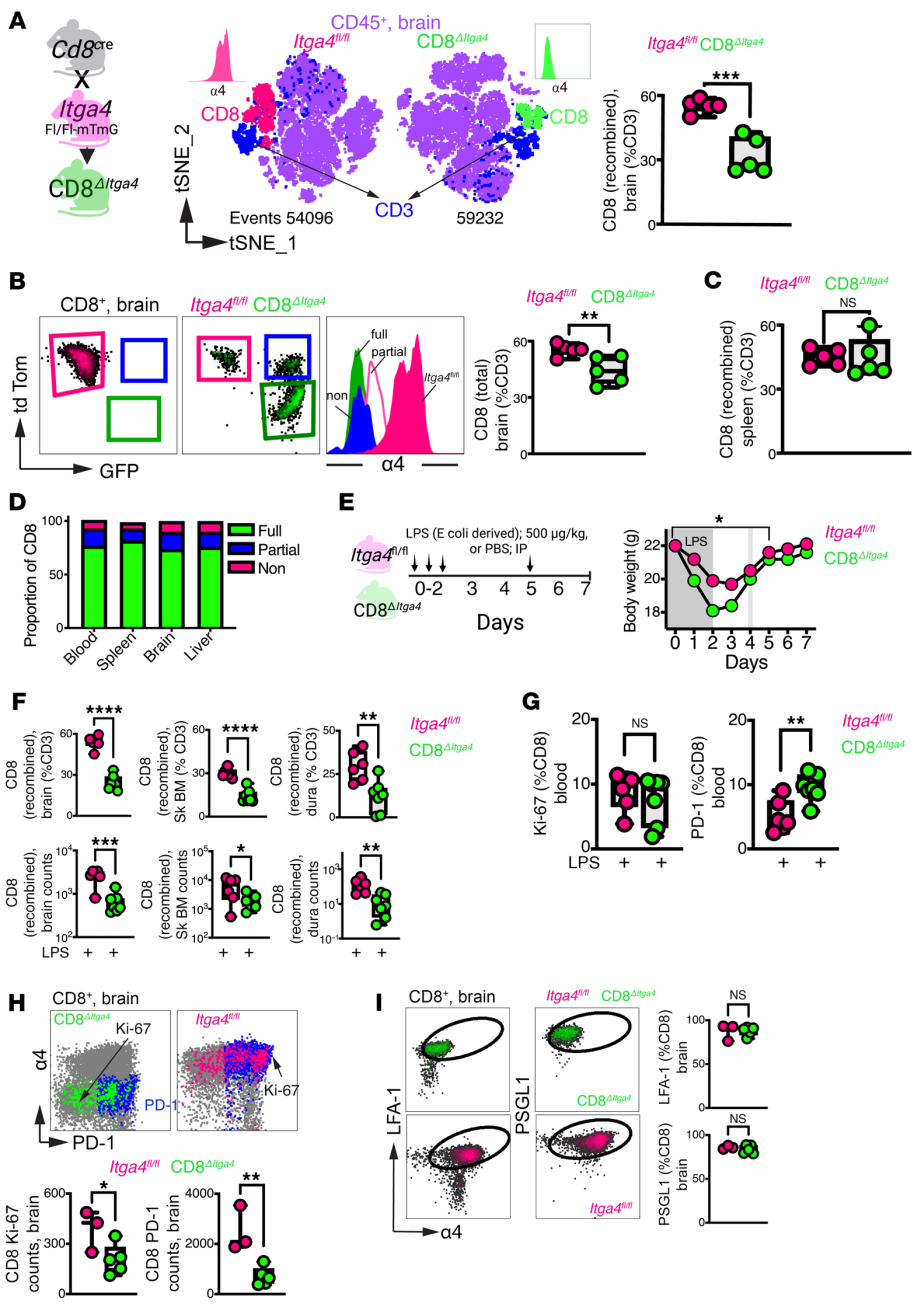
α4 Integrin regulates CD8^+^ T cell migration into the CNS and its borders. (**A**) CD8^ΔItga4^ dual-reporter system. Cre-mediated *Itga4* deletion induces tdTomato-to-GFP switch. t-SNE of CD45^+^ brain leukocytes identifies CD8^+^ T cells by reporter status. Scatterplots show frequencies of fully recombined (GFP^+^tdTomato^–^) CD8^+^ T cells in brain. (**B**) Mosaic recombination pattern in CD8^ΔItga4^ mice and corresponding reduction in total brain CD8^+^ T cell frequencies. (**C**) CD8^+^ T cell frequencies in spleen. (**D**) CD8^+^ T cell recombination state distribution across blood, spleen, brain, and liver. (**E**) LPS-induced systemic inflammation (i.p.) and body weight loss from days 1–5 after LPS. (**F**) Reduced accumulation of fully recombined CD8^+^ T cells in brain parenchyma, skull marrow, and dura, despite preserved blood frequencies. (**G**) Ki-67 and PD-1 expression on circulating CD8^+^ T cells after LPS. (**H**) Total Ki-67^+^ and PD-1^+^CD8^+^ T cell numbers in brain. (**I**) LFA-1 and PSGL-1 expression on brain-infiltrating CD8^+^ T cells. *n* = 3–5 animals per group from ≥3 independent experiments. *P < 0.05; **P < 0.01; ***P < 0.001; ****P < 0.0001, unpaired *t* test unless otherwise indicated; *P < 0.05 by paired comparison of day 0 to subsequent time points (**E**). Experiments are representative of at least 3 independent replicates.

To define α4 requirements for CD8^+^ T cell CNS trafficking, we analyzed CD8*^ΔItga4^* mice. In contrast to that seen in CD4*^ΔItga4^* mice, fully recombined α4-deleted CD8^+^ T cells were significantly reduced in brain parenchyma compared with that in *Itga4*^fl/fl^ controls (Figure 9A), indicating impaired steady-state CNS entry. CD8*^ΔItga4^* mice displayed mosaic recombination (70% fully, 20% partially, 10% nonrecombined; Figure 9B and Supplemental Figure 13). Despite this heterogeneity, total brain CD8^+^ T cell frequencies were reduced, supporting a cell-intrinsic requirement for α4 in parenchymal access.

Splenic CD8*^ΔItga4^* T cell frequencies were preserved (Figure 9C). Contingency analysis revealed similar proportions of recombination states across blood, spleen, brain, and liver (Figure 9D), indicating intact peripheral distribution and selective CNS trafficking defect rather than developmental impairment.

During LPS-induced neuroinflammation (Figure 9E), fully recombined CD8*^ΔItga4^* T cells failed to accumulate in brain parenchyma, dura, and skull marrow, despite normal frequencies in circulation (Figure 9F), indicating a persistent cell-intrinsic trafficking defect. Following LPS, CD8*^ΔItga4^* T cells upregulated Ki-67 and PD-1 in the blood with no differences in the former across genotypes (Figure 9G), confirming intact activation. Reduced CNS frequencies of CD8*^ΔItga4^* T cell effectors (Figure 9H) therefore reflect impaired effector recruitment rather than defective differentiation. LFA-1 and PSGL-1 expression remained unchanged (Figure 9I), suggesting no compensatory engagement of alternative adhesion pathways.

Together with these findings from CD4*^ΔItga4^* T cells, these data define a lineage- and compartment-specific role for α4 in CNS immune surveillance. α4 is dispensable for CD4^+^ T cell parenchymal entry but required for CNS border access. In contrast, CD8 effector recruitment to all CNS compartments depends on α4 at steady state and during inflammation. This framework explains the macaque phenotype, where preserved CD4 entry coincides with impaired CD8 access, compromising antiviral control in the inflamed brain.

## Discussion

Our macaque and mouse studies establish 3 principal findings. First, α4 integrin is dispensable for CD4^+^ T cell entry into the brain parenchyma but required for efficient CD8^+^ Tem recruitment and T cell skull bone marrow access. Second, α4 blockade alters CNS immune composition and spatial organization, preserving viral target CD4^+^ T cells while impairing CD8 effector positioning. Third, alternative adhesion and chemokine pathways permit partial T cell entry but fail to restore coordinated antiviral surveillance. These data establish α4 integrin as a context-dependent regulator of CNS immune positioning that selectively controls CD8^+^ T cell surveillance during SIV infection, rather than a universal gatekeeper of T cell brain access.

The reported paradoxical effects of α4 blockade on CNS viral control reflect experimental differences. In SIV-infected macaques, α4 blockade followed by CD8 depletion reduced cortical vRNA (30), whereas CD8-depleted CD4*^ΔItgα4^* mice failed to control vaccinia despite preserved Th17 entry (45). These divergent outcomes likely stem from distinct viral tropism, inflammation kinetics, and lymphopenia-induced CD4^+^ T cell proliferation. Critically, both studies confound α4 integrin trafficking function with CD8 depletion. This context dependence extends to autoimmune models: prophylactic α4 blockade in EAE prevents Th1 but not Th17 entry (46–48), yet during active disease with compromised CNS barrier integrity, it enhances Th1 infiltration exacerbating pathology (28). By maintaining intact CD8 populations, our study isolates the specific contribution of α4 integrin to immune positioning and antiviral coordination.

Single-cell and spatial analyses demonstrate that α4 blockade disrupts multiple layers of CNS immune coordination. CellChat inference identified reduced CD8-to-monocyte signaling under blockade, indicating impaired spatial proximity or contact-dependent communication (15, 49). VCAM-1 on activated microglia engages with α4-expressing T cells; blocking this axis may impair immune synapse formation (50). The functional consequences are evident in failed viral containment in the gm. Oral α4β1/α4β7 blockade at SIV infection increases vRNA at setpoint (31), and in the current study, α4 blockade failed to reverse SIV-induced microglial simplification. These findings parallel clinical observations in natalizumab-treated patients with MS, who exhibit increased circulating Th1 and Th17 cells with elevated cytokines (25, 27, 51), a pattern consistent with immune redistribution and loss of tissue-level coordination rather than systemic immunosuppression.

Our data implicate compensatory CNS trafficking mechanisms in the absence of α4 integrin. In mice, α4-deficient CD4^+^ T cells upregulated PSGL-1 following systemic inflammation, consistent with selectin-mediated adhesion supporting CNS entry (52, 53). PSGL-1 and αLβ2 can facilitate T cell trafficking when α4 is absent (54), but these pathways do not restore effective immune coordination. Residual CD8^+^ T cell entry despite effector reduction suggests additional adhesion or chemokine-dependent routes. Whether compensatory trafficking alters effector function remains unresolved, though PSGL-1 engagement can restrain antiviral function (55), suggesting alternative routes may permit access at the cost of function.

The present studies have several limitations that warrant consideration. In macaques, prophylactic α4 blockade with CNS analyses at 3 weeks after infection precluded assessment of eclipse phase seeding or long-term outcomes under antiretroviral therapy. Whether CD8 trafficking recovers with waning antibody levels was not examined; clinical evidence of prolonged α4 receptor occupancy (56) suggests recovery may lag behind antibody decline. The CD4*^ΔItga4^* and CD8*^ΔItga4^* mouse models enabled lineage-specific analysis but did not recapitulate systemic immune redistribution from therapeutic antibody blockade. Compensatory trafficking via PSGL-1, LFA-1, and CXCR3 was not directly tested through blockade or genetic deletion. Finally, antigen-specific T cell function was not measured in situ, and myeloid and NK cell functional states were not examined in depth.

In summary, α4 integrin functions as a context-dependent organizer of CNS immunity rather than a simple entry checkpoint. Dispensable for CD4^+^ T cell access during systemic inflammation, α4 is essential for effective CD8 effector positioning and coordinated antiviral surveillance. Its blockade permits early viral seeding while disrupting the spatial organization required for immune control. As immunomodulatory therapies for neuroinflammation advance, rational design must distinguish pathogenic from protective inflammation and preserve the adaptive immune access required for antiviral defense.

## Methods

### Sex as a biological variable.

Murine studies included both sexes. In rhesus macaque studies, all animals were female except for 1 male in the IgG-treated group. Females were selected owing to greater susceptibility to HIV-associated neuroinflammation and to reduce variability in infection kinetics.

### Rhesus macaques.

Colony-bred rhesus macaques (Macaca mulatta; *n* = 8, ages 10–20 years; median age of α4 treated, 14 years, median age of IgG treated; 14 years, median age of SIV control, 15 years) were housed at California National Primate Research Center (CNPRC) under Association for Assessment and Accreditation of Laboratory Animal Care (AAALAC) and Animal Welfare Act standards. Full details are provided in Supplemental Table 1.

### SIVmac251 infection.

Macaques were infected intravenously with 10^4^ TCID_50_ of SIVmac251 (2017 CNPRC stock, propagated in rhesus PBMCs) reconstituted in RPMI (4:1 ratio) to 500 μL final volume.

### α4 Integrin blockade.

Animals received effector-silenced anti-α4 mAb (AB_2716326) or rhesus IgG1 isotype control (AB_2716330) from the Nonhuman Primate (NHP) Reagent Resource Center. Animals were randomized and dosed intravenously (25 mg/kg) every 10 days for 3 infusions (week –1 to week 3 relative to infection). Infusions were preceded by diphenhydramine and administered as slow bolus (1–2 mL/min).

### Mice.

*Itga4 ^fl/fl^* mice (49, 57, 58), a gift from Thalia Papayannopoulou (University of Washington, Institute for Stem Cell & Regenerative Medicine, Seattle, Washington, USA). *Itga4*^fl/fl:mT/mG^ mice were generated by crossing *Itga4^fl/fl^* mice to ROSA^mT/mG^ mice (The Jackson Laboratory, strain:007676). *Itga4*^fl/fl:mT/mG^ mice were crossed with Cd4-Cre or Cd8-Cre mice (The Jackson Laboratory; 022071 and 008766) to generate CD4*^Cre^Itga4*^fl/fl:mT/mG^ (CD4*^ΔItga4^*) and CD8*^Cre^Itga4*^fl/fl:mT/mG^ (CD8*^ΔItga4^*) mice; littermate *Itga4^fl/fl^* mice served as controls. C57BL/6J mice were from The Jackson Laboratory. Both sexes (12–24 weeks old) were used. Full details are provided in Supplemental Table 2.

### LPS treatment.

Mice received i.p. LPS (500 μg/kg; *E*. *coli* O111:B4; Sigma-Aldrich, L2630) on days 0, 1, 2, and 5 (days 3–4 omitted), prepared at 250 μg/mL in PBS. Controls received matched PBS volumes. Mice were euthanized on day 7.

### Specimen collection and processing (macaque).

Animals were euthanized by a team of trained pathologists under biosafety level 2 conditions. Animals were deeply anesthetized with ketamine hydrochloride (25 mg/kg, intramuscular) and injected with a lethal dose of sodium pentobarbital (20–35 mg/kg, intravascular). Before the onset of cardiac arrest and while the animal was receiving mechanical ventilation, a thoracotomy was performed, and a cannula connected to a peristaltic pump was inserted into the left ventricle. Animals were then perfused with room temperature saline for 5 minutes, followed by 5–7 minutes of cold saline (at a flow rate of 220 mL/min). Plasma, serum, and PBMCs were collected as previously described (3). CSF was obtained via the foramen magnum and screened for blood contamination by visual inspection and Hemastix (Siemens) per the manufacturer’s instructions. Brains were harvested and hemisected, and the left hemisphere was sectioned into 6-mm-thick coronal blocks. Brain blocks were fixed by immersion in a fresh solution of 4% formaldehyde with 0.125% glutaraldehyde in phosphate buffer (pH 7.4) for 48 hours at 4°C. Blocks were stored in PBS containing 0.1% sodium azide at 4°C. For each animal, 1 rostral block (containing the PFC) and 1 caudal block (containing the Hp) were cryoprotected in progressive sucrose solutions (10%, 20%, and 30%), frozen to –20°C, and sectioned into 6 series of 30-�m-thick coronal sections using a sliding microtome coupled with a freezing stage. Sections were then individually collected and stored free-floating in a glycerol-ethylene glycol antifreeze solution at –20°C until use. Lymphoid- and CNS-associated tissues were collected and processed for mononuclear cell isolation using collagenase IV digestion and enriched using a 21/75% Percoll density gradient.

### Specimen collection and processing (mice).

Intracardiac perfusion was performed by inserting a butterfly needle attached to a 30 mL syringe into the left ventricle, followed by incision of the right atrium. Ice-cold PBS (10–15 mL) was slowly infused until liver blanching or clear efflux indicated successful perfusion. Following intracardiac perfusion, brain, skull, dura mater, spleen, liver, and Peyer’s patches were collected. All tissues except skull were mechanically dissociated and digested with 2 mg/mL collagenase type IV (Worthington Biochemical). Skull bone marrow was obtained by grinding the skull with a mortar and pestle, followed by collagenase digestion. Lymphocytes from liver and brain were further enriched using a 30/75%Percoll (Sigma-Aldrich) density gradient.

### Flow cytometry.

Whole blood and CSF were stained fresh; necropsy tissues were stained fresh or after cryopreservation. Antibodies are listed in Supplemental Table 3. Samples were acquired on BD FACSymphony or Cytek Aurora and analyzed with FlowJo v10.10.0.

### Intracellular cytokine stimulation assay.

Aliquots of 2 million freshly isolated cells or cryopreserved cells were stimulated with SIV peptide pools or PMA/Ionomycin (eBioscience Cell Stimulation Cocktail) and incubated for 1 hour at 37°C. Brefeldin A (BD GolgiPlug) and monensin (BD GolgiStop) were added to cell suspensions and incubated at 37°C for an additional 4 hours. The remainder of the procedure was carried out as previously described (59).

### vRNA quantification.

SIV RNA and DNA levels were quantified by qRT-PCR targeting the SIV *Gag* gene, as described previously (60).

### Cell preparation for sequencing studies.

Cryopreserved mononuclear cells were thawed in complete media (RPMI or DMEM with 10% FBS, 1% L-glutamine, 1% penicillin-streptomycin), treated with DNase I (2 U/mL; Roche) and CD45-enriched using NHP CD45 microbeads (Miltenyi). Live CD45^+^ cells were sorted on BD FACSAria for scRNA-seq (2).

### scRNA-seq.

Library preparation followed the Chromium Next GEM Single Cell 3’ v3.1 protocol (10x Genomics). Sequencing and bioinformatic processing were performed as described previously (3, 61).

### Spatial transcriptomics.

FFPE hippocampal sections (5 μm) were processed per NanoString GeoMx-NGS protocols (MAN-10131-02) (62). Morphology was visualized using antibodies against CD45 (Novus), CD3 (Bio-Rad), NeuN (Millipore), and Cyto83 (Thermo Fisher). Twelve ROIs (660 × 785 μm) were selected over neuron-rich and perivascular areas. Libraries were sequenced on Illumina NextSeq 2000 and aligned using NanoString GeoMx NGS pipeline v2.1.

DCC files were processed in R (geomxtools, Bioconductor v3.2.0). ROIs were excluded if alignment <80%, saturation <40%, or raw reads <1,000. Genes above LoQ in >5% of ROIs were retained and Q3 normalized. Differential expression used linear-mixed models with group as fixed effect and animal ID as random intercept; significance required BH-corrected *P* < 0.05 and |log_2_FC|>0.5. Spatial deconvolution used spatialdecon (63) with scRNA-seq–derived reference (3). Pathway analysis used GSVA (64), against Reactome pathways; heatmaps generated with ComplexHeatmap (65).

### Bioinformatics.

Raw data were aligned to Mmul_10 using Cell Ranger v6.0 (10x Genomics). Cells with <10% mitochondrial reads and genes in >10 cells were retained. Data were log normalized; UMAP visualization and graph-based clustering (resolution = 0.5) were performed. Clusters were annotated using canonical markers, differential expression, and SingleR. DGE analysis (Seurat) used adjusted *P* < 0.05 and |log_2_FC|>0.25 (BH correction). Pathway analysis used clusterProfiler v4.0. Analyses conducted in R v4.2.0.

For SIV transcript quantification, SIVmac251.RD.tf5 was added to Mmul_10 using cellranger mkref. vRNA^+^ cells were defined as SIV count >1. Interactions were excluded to prioritize high-confidence, treatment-associated changes.

### Cell-cell communication.

CellChat v1.6.1 was used with separate objects for IgG and α4 groups. Analyses were performed across Secreted Signaling, ECM–Receptor, and Cell–Cell Contact databases (https://github.com/jinworks/CellChat). Cross-condition comparisons used mergeCellChat and rankNet (https://rdrr.io/github/sqjin/CellChat/man/rankNet.html).

### In situ hybridization and CD3 immunofluorescence.

In situ hybridization was performed using a modified RNAscope protocol (66, 67), with custom SIVmac239 probe (Gag, Pol, Nef) and a RNAscope 2.5 HD Detection Kit (ACD). FFPE sections (4 μm) were pretreated, hybridized (2 h, 40°C), and detected with DAB or TSA Vivid fluorophore 520 (catalog 323271, ACD/Bio-Techne). For combined staining, slides were incubated with rat anti-CD3 (Abcam, 1:100) followed by Alexa Fluor 568 secondary antibody (Invitrogen) and DAPI. Images were acquired on a Zeiss LSM800 or Imager Z2. Controls included dapB probe and uninfected tissues.

### Immunohistochemistry.

Fluorescence immunohistochemistry was performed as described previously (68). Three sections/block/animal underwent citrate buffer antigen retrieval (pH 6.1, 60°C, 30 min), blocking (5% NGS, 5% NDS, 5% BDA in PBS/0.3% Triton-X, 2 h), and primary antibody incubation (48 h, 4°C): IBA1 (Wako 019-19741, rabbit, 1 μg/section), HLA-DR (Invitrogen, 14-9956-82, mouse, 2.5 μg/section), and NeuN (Synaptic Systems, 266004, guinea pig, 1 μL/section). Secondary antibodies included Alexa Fluor 488 anti-rabbit, 555 anti-mouse, 647 anti-guinea pig (Invitrogen). Sections were counterstained with DAPI and mounted with ProLong Gold.

### Microscopy.

Images were acquired on a Zeiss AxioImager Z2/LSM800 confocal microscope (20× objective, 1,024 p, 16-bit). Four images/slide were acquired from PFC Layer III and underlying wm; hippocampal images included CA1 (4 images), CA3 (2 images), and CA4/hilus (2 images) fields. Experimenters were blinded to IBA1/HLA-DR channels during selection. Acquisition parameters are detailed in Supplemental Methods.

### Image segmentation.

Processing used a custom Python 3.11 pipeline (scikit-image, numpy, scipy). Four-channel OME-TIFFs (HLA-DR, IBA1, DAPI, NeuN) were processed per-scene. Z-band selection was DAPI-based using Otsu thresholding. DAPI masks were generated using the Triangle method with morphological refinement; objects <20 μm³ were removed. IBA1 segmentation combined Frangi-filtered process maps with Otsu-thresholded soma masks. HLA-DR used adaptive normalization, rolling-ball background subtraction, and Yen-based hysteresis thresholding. Full pipeline details are provided in Supplemental Methods.

### Microglia morphological analysis.

3D reconstruction from IBA1 masks quantified soma volume, arbor length, branch points, and endpoints using custom Python workflow. Spatial metrics included cell density, Clark-Evans R index, Hopkins H statistic, and Ripley’s L(r)–r function (5–150 μm radii). Methods are detailed in Supplemental Methods.

### Statistics.

Wilcoxon’s signed-rank test was used for paired analyses; Mann-Whitney *U* test was used for unpaired comparisons. Tests were performed in GraphPad Prism v10.5.0; *P* < 0.05 was considered significant. For sequencing analyses, Benjamini-Hochberg correction was applied. For microglial assessments, data meeting parametric assumptions (Brown-Forsythe test, |skewness|≤1, |kurtosis|≤3) were analyzed by 1-way ANOVA with Tukey’s HSD; otherwise Kruskal-Wallis with Mann-Whitney *U* and Holm’s correction was used. Transformations (square root or log[1+*x*]) were applied when appropriate.

### Study approval.

All macaque procedures were approved by the IACUC of the University of California, Davis (protocols 22379, 22261, 22787, 22033, 23363). Mouse experiments were approved by the IACUC of the University of Pittsburgh (protocols 23093137, 24074389). All procedures were conducted in accordance with AAALAC guidelines.

### Data and materials availability.

Supporting data values are provided in the Supporting Data Values file. scRNA-seq data are accessible at GEO (GSE221815); spatial transcriptomics data are accessible at GEO (GSE279903).

## Author contributions

PBP, SRE, YSL, AV, RPR, GBD, JHM, RR, and SSI designed the studies. JHM, RR, and SSI supervised experiments. PBP, SRE, YSL, AV, RPR, D Rossmiler, JK, RS, SO, CTE, KS, ZMM, and RR performed experiments. PBP, SRE, YSL, GBD, DB, ZMM, AP, DN, D Rajasundaram, SSI analyzed data. PBP, SRE, GBD, and SSI wrote and revised the manuscript. All authors read, edited, and approved the final manuscript. Co–first author order was determined by contribution.

## Conflict of interest

DN is an employee and shareholder of NanoString Technologies/Bruker Spatial Biology.

## Funding support

This work is the result of NIH funding, in whole or in part, and is subject to the NIH Public Access Policy. Through acceptance of this federal funding, the NIH has been given a right to make the work publicly available in PubMed Central.

- NIH grants K01OD023034 (to SSI), RF1AG06001 (to SSI and JHM), R56AI150409 (to SSI), 1R01AI187016-01 (to SSI), K01DK110264 (to RR), and R01DK124351 (to RR).
- National Institute of Allergy and Infectious Diseases grants U24 AI126683 (to NHPRR) and HHSN261201500003I and 75N91019D00024 (to the Quantitative Molecular Diagnostics Core, AIDS and Cancer Virus Program, Frederick National Laboratory).

## Supplementary Material

Supplemental data

Supporting data values

## Version 1 02/24/2026

In-Press Preview

## Version 2 04/15/2026

Electronic publication
