# Supplementary material for: α4 Integrin blockade impairs CD8+ T cell neuroimmune surveillance following SIV infection
Source: J Clin Invest. 2026 Feb 24;136(8):e198730. doi: 10.1172/JCI198730 (PMC13078879; doi:10.1172/JCI198730)
Supplement: Supplemental data [file jci-136-198730-s264.pdf]

**Supplemental Data**

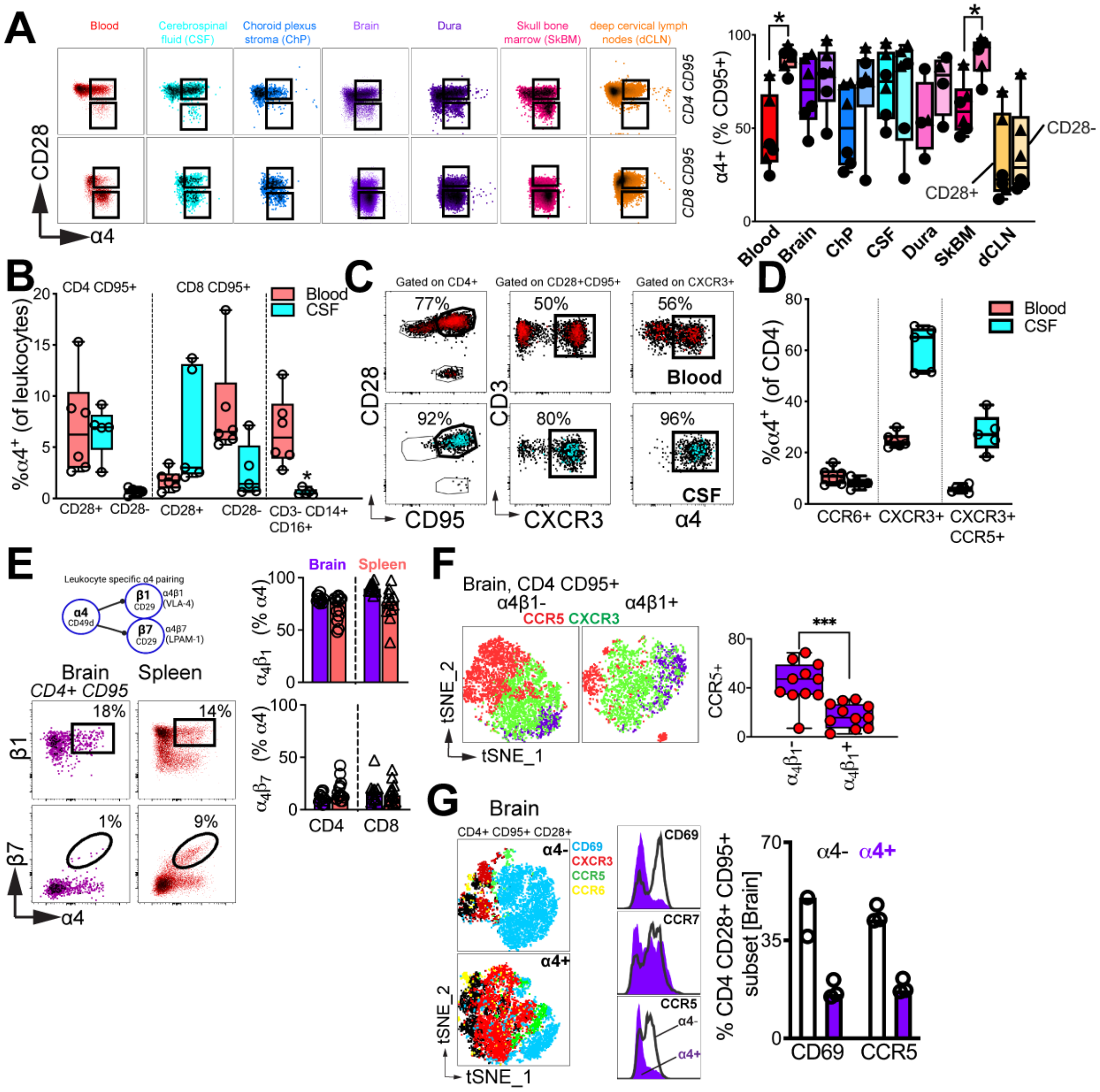

**Supplementary Figure 1. Integrin and chemokine receptor profiling of α4⁺ T cells across CNS and systemic compartments. (A)** α4 integrin expression in $CD28^-$ and $CD28^+$ T cell subsets (n=7 animals across 6 tissues) across indicated tissues; cells are color-coded by tissue of origin in both flow plots and box plots. (**B**) Frequencies of $\alpha4^+$ $CD28^+$ and $CD28^-$ CD4 and CD8 T cells compared with $\alpha4^+$ monocytes in blood and cerebrospinal fluid (CSF). (**C**) Representative flow cytometry gating strategy for $CD28^+$ $CD95^+$ T cells in blood (red, top) and CSF (blue, bottom), with CXCR3 expression and corresponding α4 integrin expression shown on the $CXCR3^+$ subset. (**D**) Distribution of $CXCR3^+$ ($T_h1$) and $CCR6^+$ ($T_h17$) subsets and target $T_h1$ cells ($CCR5^+$ $CXCR3^+$) within $\alpha4^+$ CD4 T cells in blood and CSF. (**E**) Co-expression of β1 and β7 integrin chains on $\alpha4^+$ T cells in brain and spleen. (**F**) Chemokine receptor expression profiles of $\alpha4\beta1^-$ and $\alpha4\beta1^+$ CD4 T cells (n=11) in the brain. (**G**) Expression of CD69, CCR7, and CCR5 on $\alpha4^-$ and $\alpha4^+$ CD4 T cells in the brain. *$p < 0.05$, two-tailed t-test **(A)**; ***$p < 0.001$, two-tailed t-test **(F)**. ChP, choroid plexus stroma; SkBM, skull bone marrow; dCLN, deep cervical lymph nodes.

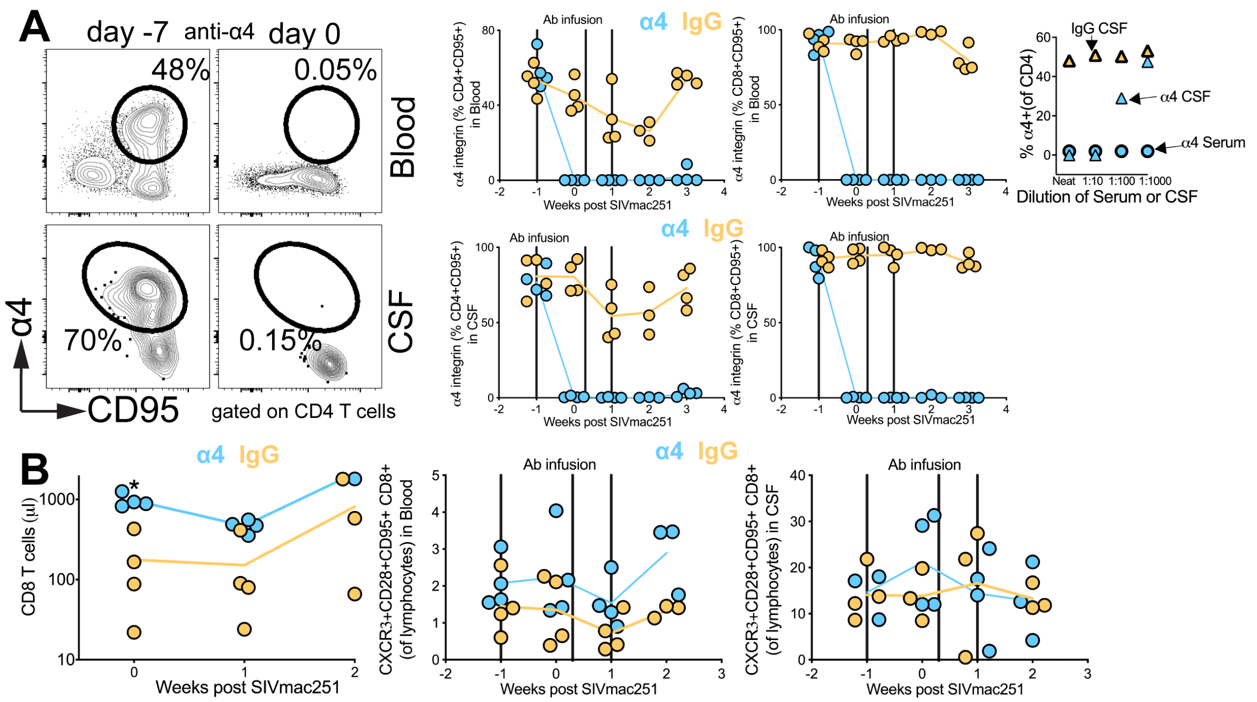


**Supplementary Figure 2. α4 integrin blockade and T cell subset kinetics in blood and cerebrospinal fluid. (A)** α4 integrin receptor occupancy analysis demonstrating sustained blockade on T cells in blood and cerebrospinal fluid (CSF) following α4 antibody administration. Inset shows ex vivo receptor occupancy assay, in which pre-infusion PBMCs were incubated with serum (circles) or CSF (triangles) from α4-treated animals, resulting in loss of detectable surface α4 expression, which was recovered by dilution of CSF but not serum. CSF from IgG-treated animal shown as control. **(B)** Longitudinal frequencies of total CD8 T cells and $CXCR3^{+}$ CD8 T cell subsets in blood and CSF over time following α4 blockade. *$p < 0.05$, one-tailed unpaired Mann–Whitney test; n = 4 per group.

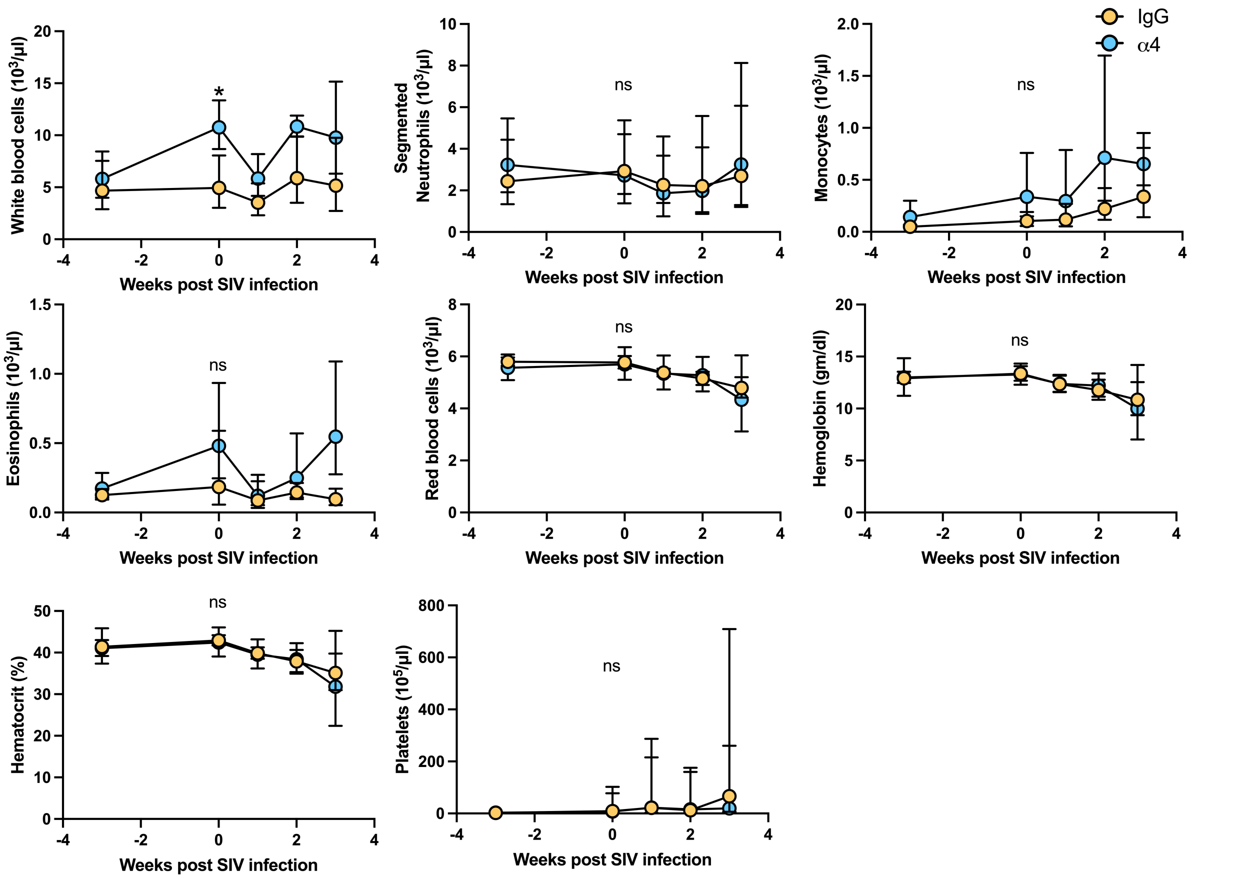


**Supplementary Figure 3. α4 integrin blockade does not alter innate immune cell frequencies or hematologic parameters.** Longitudinal measurements of circulating monocytes, neutrophils, eosinophils, red blood cells, hemoglobin, hematocrit, and platelets in α4-treated and IgG-treated animals. Data are shown as geometric mean with standard deviation. n = 4 animals per group.

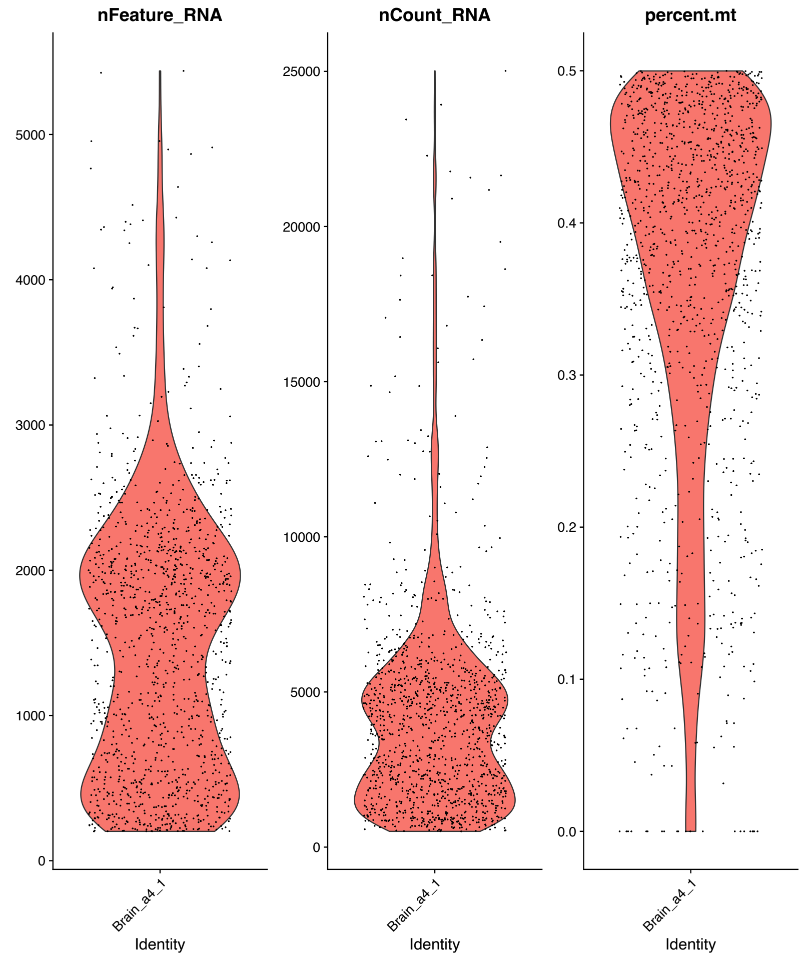


**Supplementary Figure 4A. Single-cell RNA sequencing quality control metrics for a representative brain sample (α4 replicate A4_1, n = 4 total samples).** Violin plots display the distribution of detected genes per cell (nFeature_RNA), total transcript counts per cell (nCount_RNA), and the proportion of mitochondrial transcripts (percent.mt) across $CD45^+$ brain immune cells from one α4-treated animal (Brain A4_1). Each point represents an individual cell. These metrics were used to assess data quality and to exclude low-quality cells prior to downstream clustering and differential expression analyses.

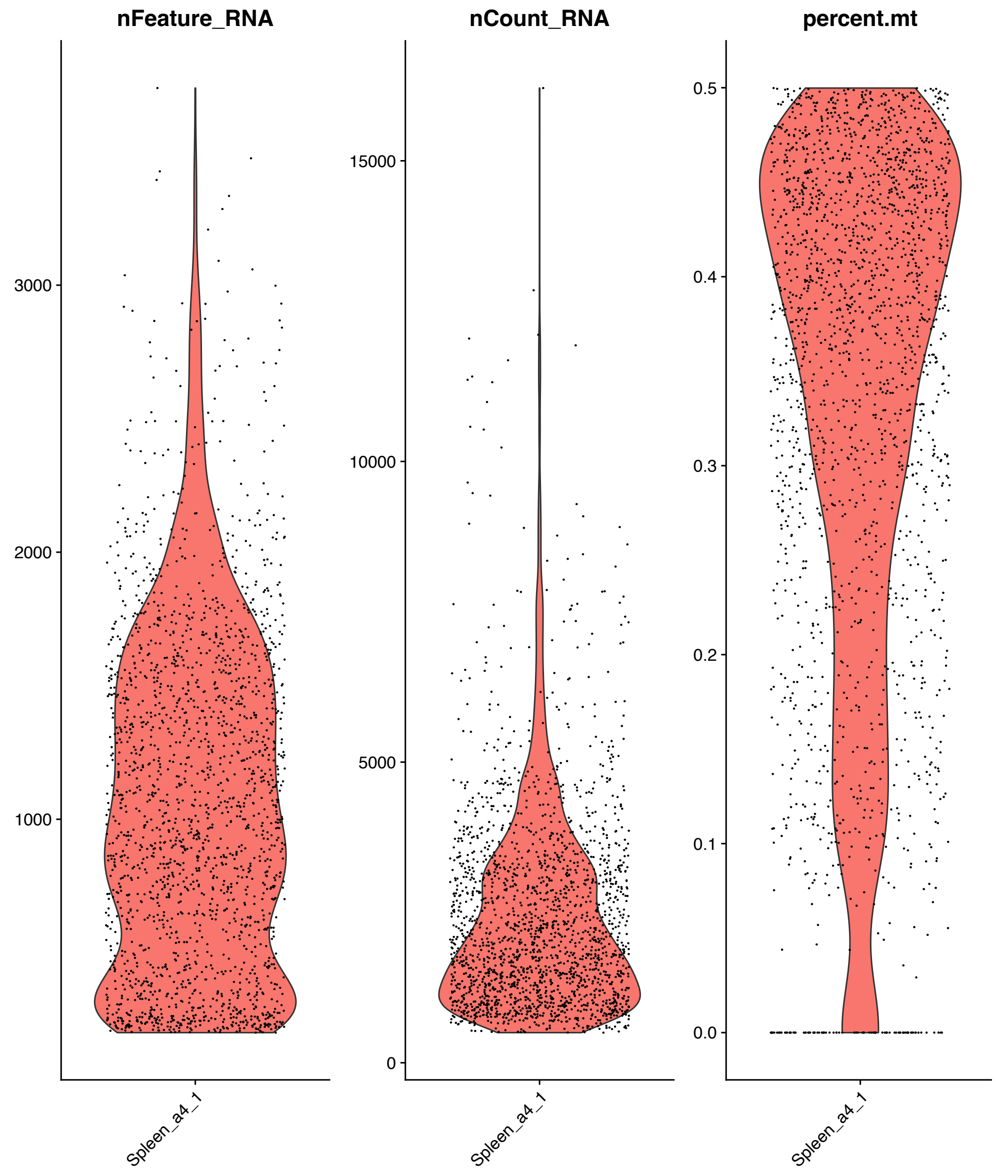


**Supplementary Figure 4B. Single-cell RNA sequencing quality control metrics for a representative spleen sample (α4 replicate A4_1, n = 4 total samples).** Violin plots display the distribution of detected genes per cell (nFeature_RNA), total transcript counts per cell (nCount_RNA), and the proportion of mitochondrial transcripts (percent.mt) across $CD45^+$ spleen immune cells from one α4-treated animal (Spleen A4_1). Each point represents an individual cell. These metrics were used to assess data quality and to exclude low-quality cells prior to downstream clustering and differential expression analyses.

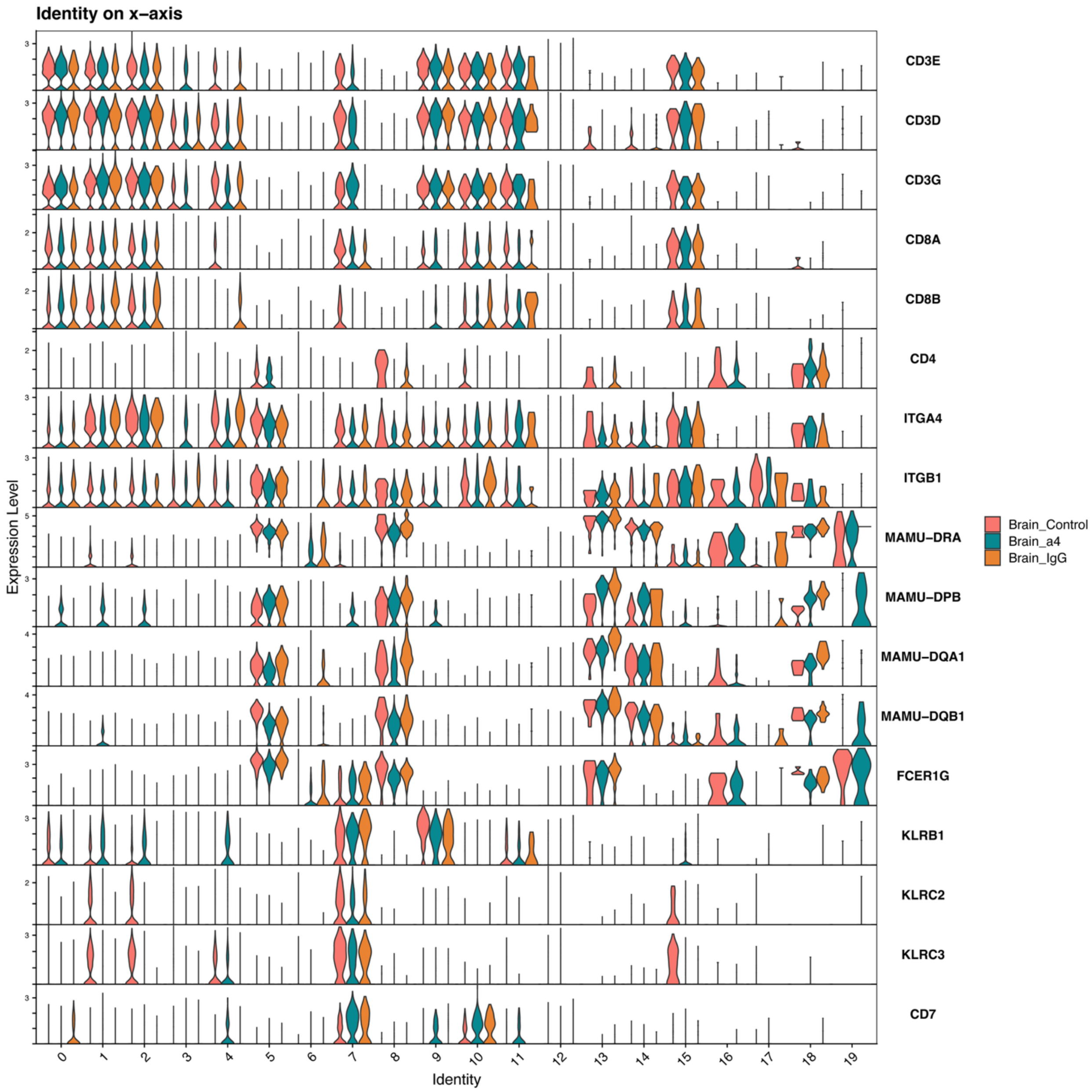


**Supplementary Figure 5. Distribution of *ITGA4* expression across brain immune subclusters in control, α4-treated, and IgG-treated animals.** *ITGA4* gene expression is shown across 20 annotated brain immune subclusters, displayed separately for control (n = 2), IgG-treated (n = 4), and α4-treated (n = 4) animals (see Figure 2C for consolidated expression across all conditions). Control data were previously published (Ref #3).

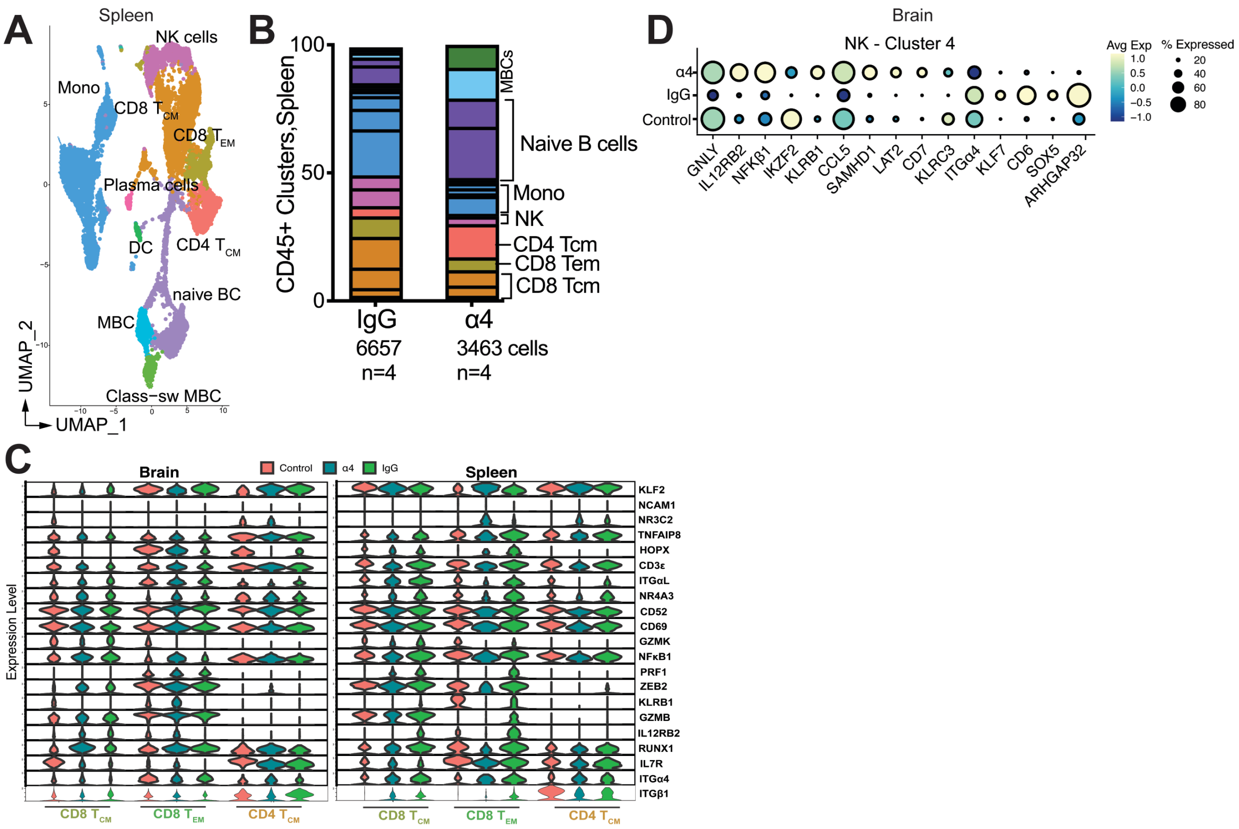


**Supplementary Figure 6. Distribution of *ITGA4* expression across spleen immune populations.** (**A–C**) UMAP visualizations of spleen immune populations, including B cell clusters absent from the brain dataset, shown in control (n = 2), IgG-treated (n = 4), and α4-treated (n = 4) animals. (**D**) Dot plot showing transcriptional features of the NK cell cluster in the brain. Control data were previously published (Ref #3).

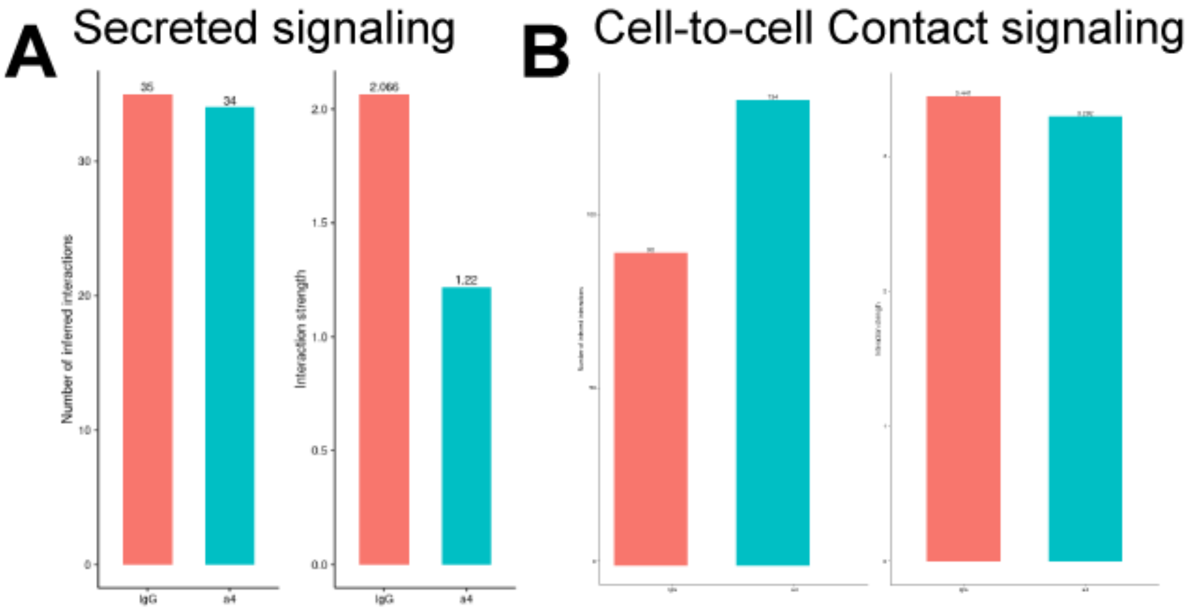


**Supplementary Figure 7. Inferred secreted and contact-mediated signaling under α4 integrin blockade. (A)** CellChat analysis showing reduced strength, but not number, of predicted secreted signaling interactions following α4 blockade. **(B)** In contrast, contact-mediated signaling exhibits an increased number of interactions under blockade, while overall interaction strength remains comparable between groups. n = 3 per group.

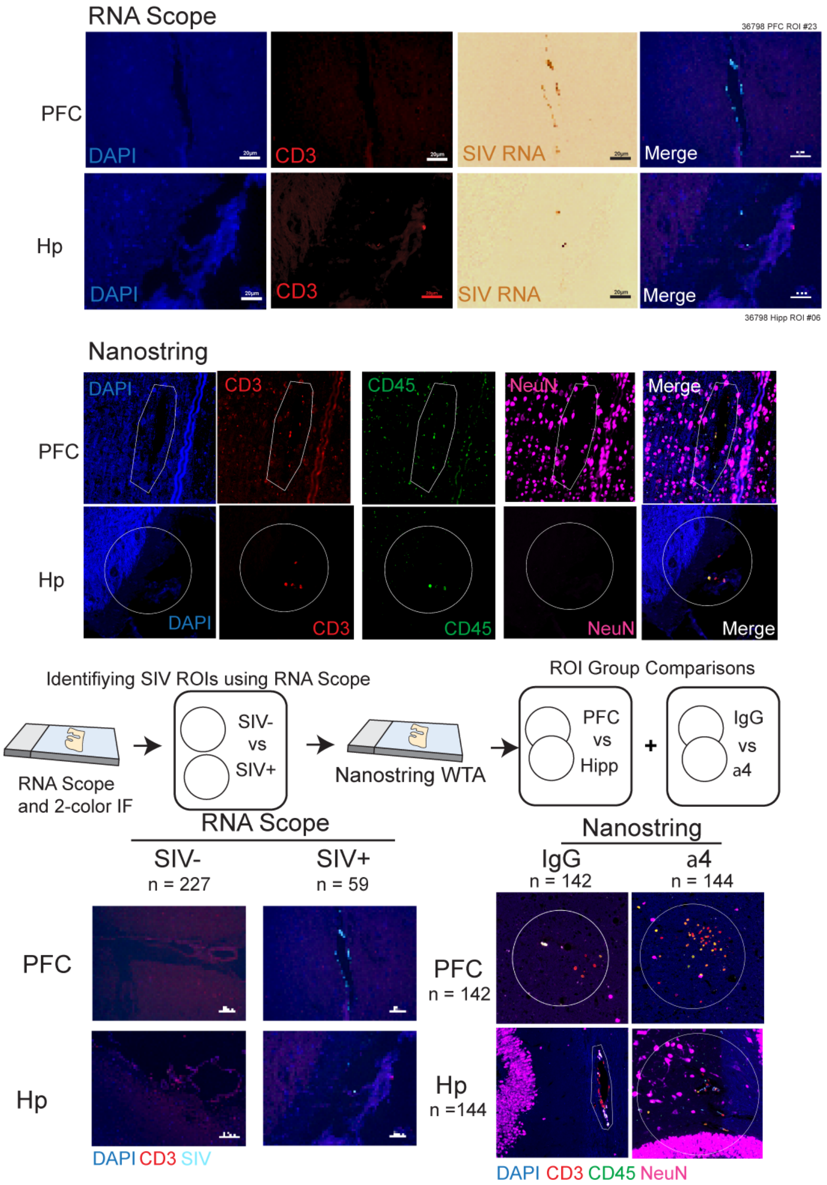


**Supplementary Figure 8. Spatial ROI selection and sample overview for Nanostring transcriptomic analysis.** Representative tissue sections showing SIV RNA$^{+}$ and SIV$^{-}$ regions identified by RNAscope in situ hybridization, which were used to guide region of interest selection for downstream NanoString whole transcriptome analysis (WTA). n = 3 per group, Scale bar: 20μm.

The RNAscope image in the S8 top panel is reused in the S8 lower left panel (SIV$^{+}$/HP). All analyses and quantification were performed on independent biological replicates.

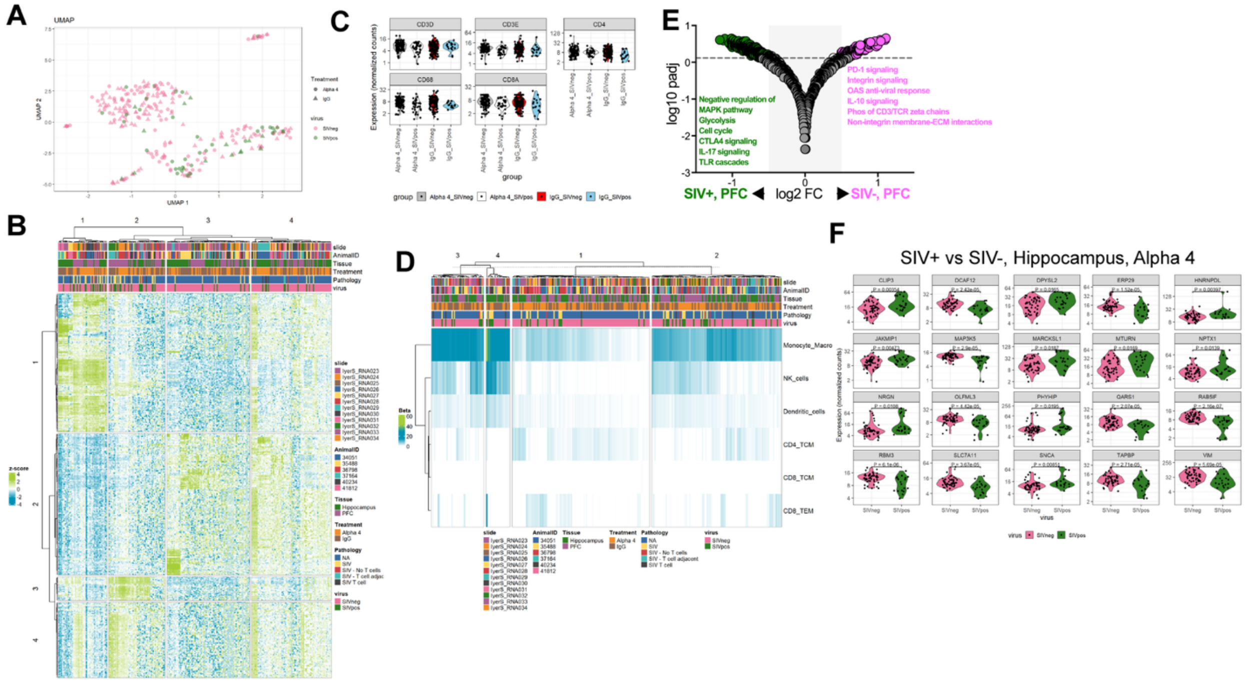


**Supplementary Figure 9. SIV RNA status drives spatial clustering and immune cell localization. (A–B)** Dimensionality reduction of NanoString whole transcriptome data showing region of interest clustering primarily by SIV RNA status. **(C)** Spatial expression of immune marker transcripts (CD3, CD4, CD8, CD68) confirming localization of T cell and myeloid populations across regions of interest. **(D)** Composition of the sc-RNA-seq-derived reference used for spatial deconvolution, showing marker genes defining CD4 T cell, CD8 T cell, NK cell, dendritic cell, and myeloid clusters used to estimate cell type proportions in spatial segments. **(E)** Volcano plot displaying the distribution of differentially expressed genes with adjusted P values. **(F)** Violin plots showing differences in gene expression across SIV- versus SIV+ hippocampal regions in α4-treated animals. n = 3 per group.

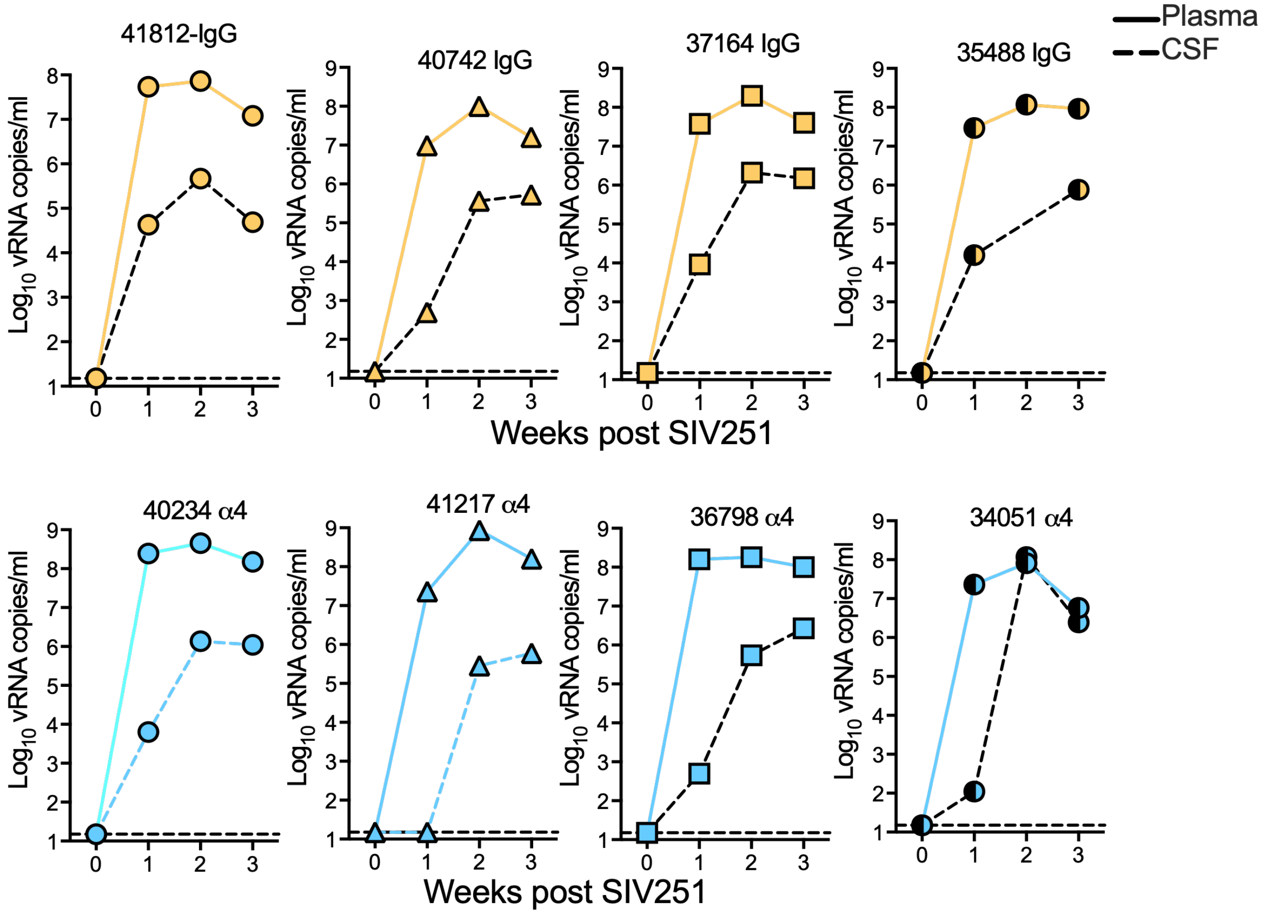


**Supplementary Figure 10. Individual viral kinetics in plasma and cerebrospinal fluid by treatment group.** Longitudinal plasma (solid line) and cerebrospinal fluid (CSF, dashed line) viral(v) RNA levels for individual animals from weeks 1 to 3 post-infection in α4-treated and IgG-treated groups.

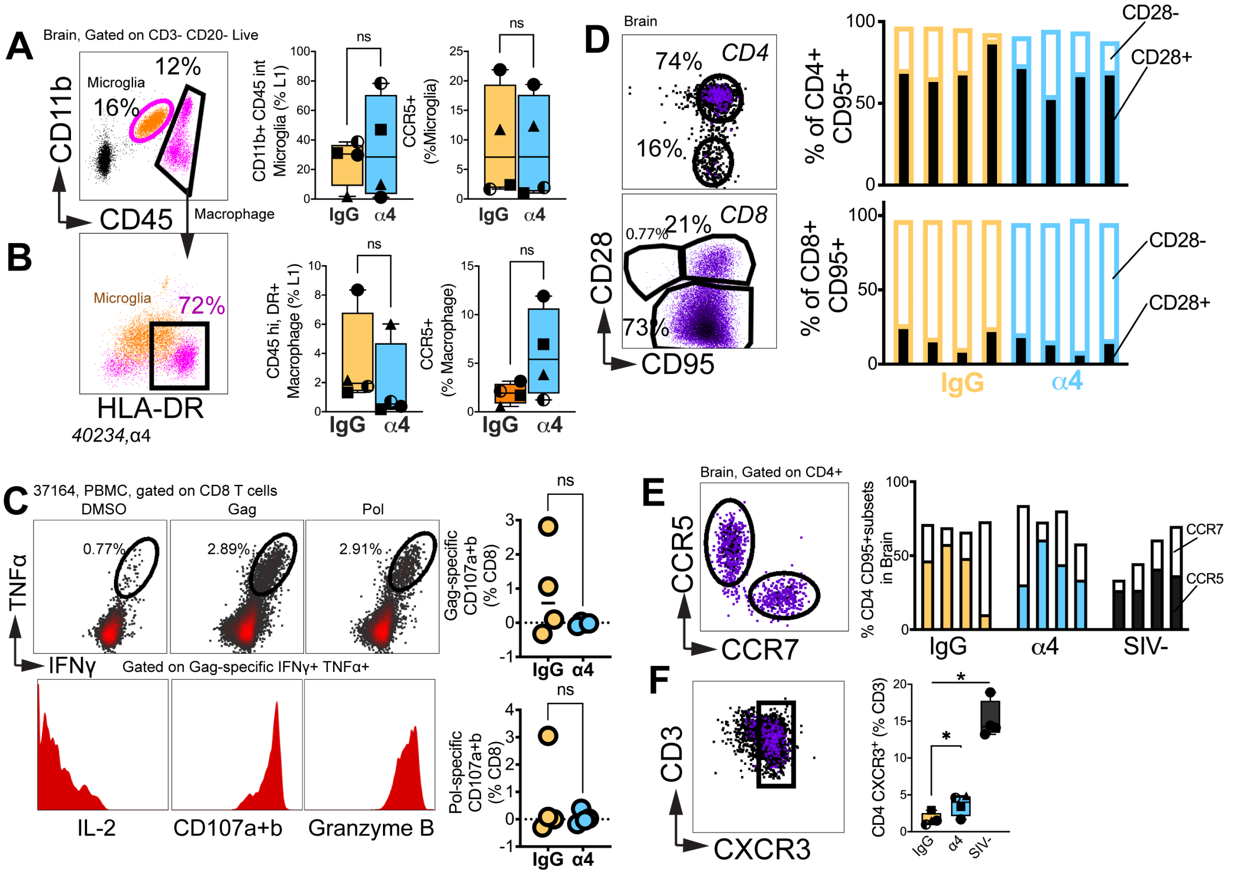


**Supplementary Figure 11. Myeloid and T cell subset characterization in brain tissue following α4 integrin blockade in macaques.** (**A–B**) Quantification of microglia and macrophages in brain tissue from α4-treated and IgG-treated animals, showing no significant differences between groups. (**C**) Flow plot shows SIV-specific T cell responses to Gag and Pol peptides by ICS in blood; histograms show expression of IL-2 and cytolytic molecules on Gag-specific IFNγ+ TNFα+ CD8 T cells; scatter plots show frequencies of DMSO-subtracted Gag-and Pol-specific CD8 T cells across groups. (**D**) Expression profiles of $CD28^+$ and $CD28^-$ CD4 T cell subsets in the brain. (**E**) Expression of chemokine receptors CCR5 and CCR7 on brain-infiltrating CD4 T cells. (**F**) Frequencies of $CXCR3^+$ CD4 T cells within the brain parenchyma, showing reduced integrin-expressing subsets following α4 blockade. *$p < 0.05$, one-tailed unpaired Mann–Whitney test; n = 4 per group.

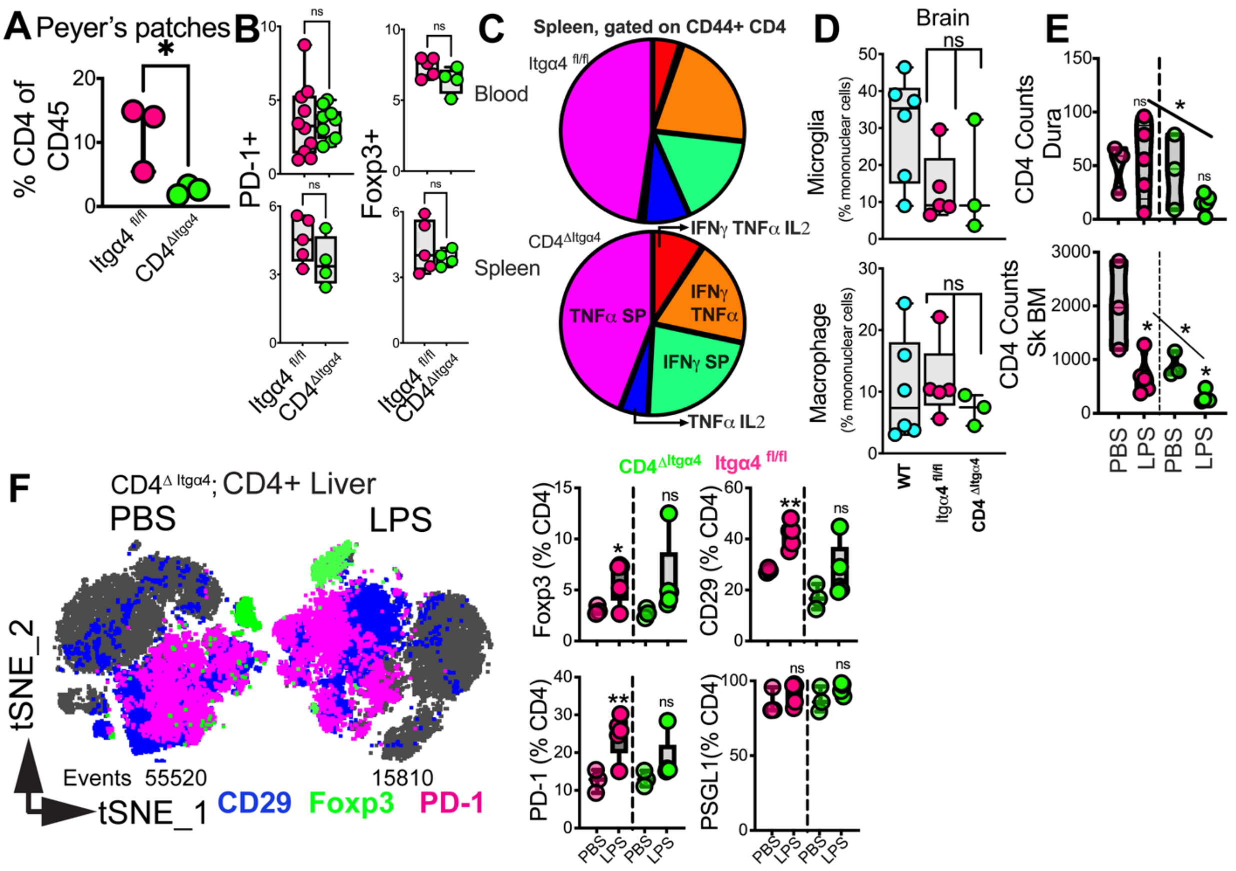


**Supplementary Figure 12. Effects of α4 integrin deletion on CD4 T cell distribution and phenotype across tissues. (A)** Reduced frequencies of CD4 T cells in Peyer's patches of CD4$^{\Delta Itg\alpha4}$ mice. (**B**) PD-1 and Foxp3 expression in circulating and splenic CD4 T cells across genotypes. (**C**) Boolean analysis of cytokine-producing CD44$^{+}$ CD4 T cells in the spleen following PMA and ionomycin stimulation. (**D**) Frequencies of brain-resident innate immune populations, including microglia and macrophages, across groups. (**E**) CD4 counts in dura and skull bone marrow. (**F**) Phenotypic profiling of liver CD4 T cells following LPS challenge, showing comparable activation and trafficking-associated marker expression across genotypes. Data represent 3–5 animals per group from ≥3 independent experiments.

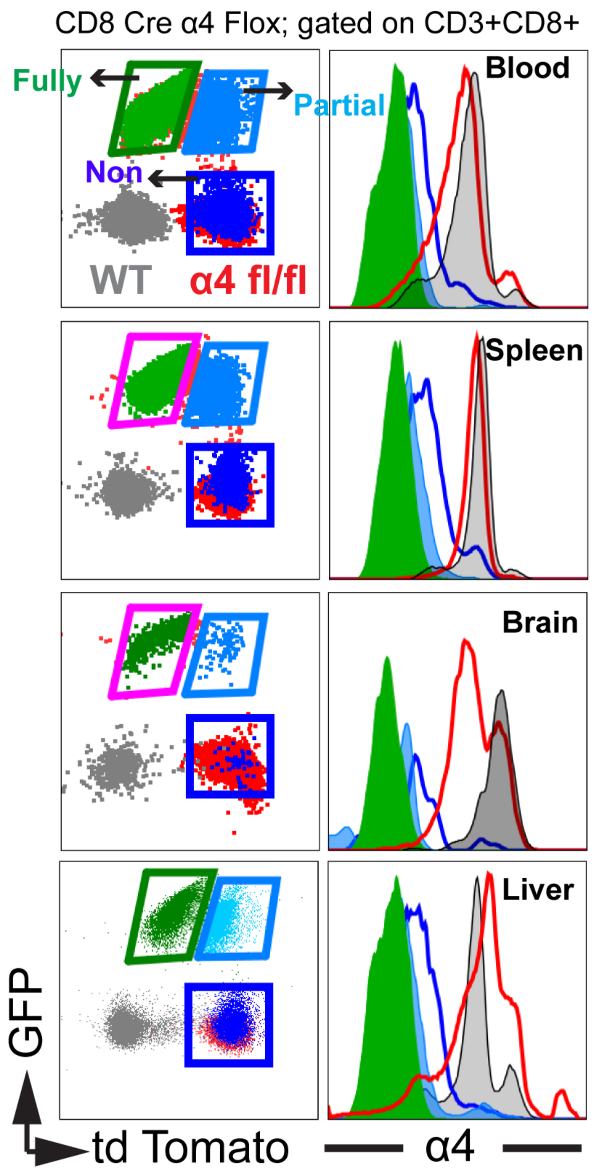


**Supplementary Figure 13. Mosaic recombination of *Itgα4* in CD8$^{\Delta Itg\alpha 4}$ mice.** Representative flow cytometry plots illustrating fully recombined (GFP$^{+}$ tdTomato$^{-}$), partially recombined, and non-recombined CD8 T cell populations in CD8$^{\Delta Itg\alpha 4}$ mice across blood, spleen, brain, and liver. Data represent 3–5 animals per group from ≥3 independent experiments.

**S1 Table. Nonhuman primate cohorts.**

| Group | Animal ID | Sex | Age (years. months) at Nx. | Weight (kg) at Nx. | Site | Virus ($1x10^4$ $TCID_{50}$) | IgG antibody* control (25mg/kg) | ART Regimen |
|---|---|---|---|---|---|---|---|---|
| α4 blockade study (n=8) | 41812 | F | 11.02 | 7.30 | CNPRC | SIVmac251 | Anti-desipramine | - |
| | 40742 | M | 12.04 | 12.00 | CNPRC | SIVmac251 | Anti-desipramine | - |
| | 37164 | F | 16.01 | 10.83 | CNPRC | SIVmac251 | Anti-desipramine | - |
| | 35488 | F | 17.04 | 10.48 | CNPRC | SIVmac251 | Anti-desipramine | - |
| | 40234 | F | 12.11 | 9.34 | CNPRC | SIVmac251 | Anti-α4 | - |
| | 41217 | F | 11.10 | 12.3 | CNPRC | SIVmac251 | Anti-α4 | - |
| | 36798 | F | 16.11 | 10.8 | CNPRC | SIVmac251 | Anti-α4 | - |
| | 34051 | F | 20.00 | 7.31 | CNPRC | SIVmac251 | Anti-α4 | - |
| Chronic 251 Cohort (n=6) | 38889[&] | F | 14.06 | 11.31 | CNPRC | SIVmac251 | - | FTC/TDF/DTG |
| | 38919 | F | 14.06 | 8.63 | CNPRC | SIVmac251 | - | FTC/TDF/DTG |
| | 36056 | F | 18.06 | 9.96 | CNPRC | SIVmac251 | - | FTC/TDF/DTG |
| | 37274 | F | 16.06 | 8.67 | CNPRC | SIVmac251 | - | FTC/TDF/DTG |
| | 39359 | M | 14.04 | 13.36 | CNPRC | SIVmac251 | - | FTC/TDF/DTG |
| | 36511[&] | F | 17.06 | 10.34 | CNPRC | SIVmac251 | - | FTC/TDF/DTG |
| Control Cohort 1 (n=4) | 38163 | F | 15.07 | 7.48 | CNPRC | - | - | - |
| | 40691 | M | 12.07 | 12.18 | CNPRC | - | - | - |
| | 38691 | F | 14.10 | 13.56 | CNPRC | - | - | - |
| | 40499 | F | 12.11 | 9.79 | CNPRC | - | - | - |
| | | | | | | | | |

Animals in the α4 blockade cohort were included throughout the study and are presented in Figures 1 through 6. Data from control cohort are shown in single-cell analysis, microscopy, and phenotype characterization in Figures 2, 5, and 6. Data from the chronic 251 cohort are shown specifically in Figure 6. SIV: Simian Immunodeficiency Virus; ART: Anti-Retroviral Therapy; FTC: Emtricitabine; TDF: Tenofovir disoproxil fumarate; DTG: Dolutegravir; CNPRC: California National Primate Research Center

*Antibody treatments were administered at 1 week prior to SIV infection and every subsequent 10-day interval at doses of 25mg/kg intravenously over the course of the study.

## S2 Table. Details of mice

| *Strain* | Age (Months) | Sex | Date assessed | Blood | Spleen | Liver | PP | Brain | Dura | Sk BM | PBS | LPS |
|---|---|---|---|---|---|---|---|---|---|---|---|---|
| *WT-MS-1* | 6 mo | M | Jan 23 2025 | ☑ | ☑ | ☑ | ☑ | ☑ | X | X | X | X |
| *WT-MS-2* | 6 mo | M | Jan 23 2025 | ☑ | ☑ | ☑ | ☑ | ☑ | X | X | X | X |
| *WT-MS-3* | 6 mo | M | Jan 23 2025 | ☑ | ☑ | ☑ | ☑ | ☑ | X | X | X | X |
| *A4 Fl-MS-1* | 6 mo | M | Jan 23 2025 | ☑ | ☑ | ☑ | ☑ | ☑ | X | X | X | X |
| *A4 Fl-MS-2* | 6 mo | M | Jan 23 2025 | ☑ | ☑ | ☑ | ☑ | ☑ | X | X | X | X |
| *A4 Fl-MS-3* | 6 mo | M | Jan 23 2025 | ☑ | ☑ | ☑ | ☑ | ☑ | X | X | X | X |
| *CD4CreA4 Fl-MS-1* | 6 mo | M | Jan 23 2025 | ☑ | ☑ | ☑ | ☑ | ☑ | X | X | X | X |
| *CD4CreA4 Fl-MS-2* | 6 mo | M | Jan 23 2025 | ☑ | ☑ | ☑ | ☑ | ☑ | X | X | X | X |
| *CD8CreA4 Fl-MS-1* | 6 mo | M | Jan 23 2025 | ☑ | ☑ | ☑ | ☑ | ☑ | X | X | X | X |
| *CD8CreA4 Fl-MS-2* | 6 mo | M | Jan 23 2025 | ☑ | ☑ | ☑ | ☑ | ☑ | X | X | X | X |
| *CD8CreA4 Fl-MS-3* | 6 mo | M | Jan 23 2025 | ☑ | ☑ | ☑ | ☑ | ☑ | X | X | X | X |
| | | | | | | | | | | | | |
| *WT-MS-1* | 7 mo | F | Feb 27 2025 | X | ☑ | ☑ | ☑ | ☑ | X | X | X | X |
| *WT-MS-2* | 7 mo | F | Feb 27 2025 | X | ☑ | ☑ | ☑ | ☑ | X | X | X | X |
| *WT-MS-3* | 7 mo | F | Feb 27 2025 | X | ☑ | ☑ | ☑ | ☑ | X | X | X | X |
| *A4 Fl-MS-1* | 7 mo | F | Feb 27 2025 | X | ☑ | ☑ | ☑ | ☑ | X | X | X | X |
| *A4 Fl-MS-2* | 7 mo | F | Feb 27 2025 | X | ☑ | ☑ | ☑ | ☑ | X | X | X | X |
| *CD4CreA4 Fl-MS-1* | 7 mo | F | Feb 27 2025 | X | ☑ | ☑ | ☑ | ☑ | X | X | X | X |
| *CD8CreA4 Fl-MS-1* | 7 mo | F | Feb 27 2025 | X | ☑ | ☑ | ☑ | ☑ | X | X | X | X |
| *CD8CreA4 Fl-MS-2* | 7 mo | F | Feb 27 2025 | X | ☑ | ☑ | ☑ | ☑ | X | X | X | X |
| | | | | | | | | | | | | |
| *A4 Fl-MS-1* | 3 mo | F | May 22 2025 | ☑ | X | X | X | X | X | X | X | X |
| *A4 Fl-MS-2* | 3 mo | F | May 22 2025 | ☑ | X | X | X | X | X | X | X | X |
| *A4 Fl-MS-3* | 3 mo | F | May 22 2025 | ☑ | X | X | X | X | X | X | X | X |
| *A4 Fl-MS-1* | 3 mo | M | May 22 2025 | ☑ | X | X | X | X | X | X | X | X |
| *A4 Fl-MS-2* | 3 mo | M | May 22 2025 | ☑ | X | X | X | X | X | X | X | X |
| *CD4CreA4 Fl-MS-1* | 3 mo | F | May 22 2025 | ☑ | X | X | X | X | X | X | X | X |
| *CD4CreA4 Fl-MS-2* | 3 mo | F | May 22 2025 | ☑ | X | X | X | X | X | X | X | X |
| *CD4CreA4 Fl-MS-3* | 3 mo | F | May 22 2025 | ☑ | X | X | X | X | X | X | X | X |
| *CD4CreA4 Fl-MS-1* | 3 mo | M | May 22 2025 | ☑ | X | X | X | X | X | X | X | X |
| *CD4CreA4 Fl-MS-2* | 3 mo | M | May 22 2025 | ☑ | X | X | X | X | X | X | X | X |
| *CD4CreA4 Fl-MS-3* | 3 mo | M | May 22 2025 | ☑ | X | X | X | X | X | X | X | X |
| | | | | | | | | | | | | |
| *CD4CreA4 Fl-MS-1* | 3-4mo | F | April 20 2025 | ☑ | ☑ | ☑ | ☑ | ☑ | ☑ | ☑ | X | ☑ |
| *CD8CreA4 Fl-MS-1* | 3-4mo | F | April 20 2025 | ☑ | ☑ | ☑ | ☑ | ☑ | ☑ | ☑ | X | ☑ |
| *CD8CreA4 Fl-MS-2* | 3-4mo | F | April 20 2025 | ☑ | ☑ | ☑ | ☑ | ☑ | ☑ | ☑ | X | ☑ |
| *A4 Fl-MS-1* | 3-4mo | F | April 20 2025 | ☑ | ☑ | ☑ | ☑ | ☑ | ☑ | ☑ | X | ☑ |
| *A4 Fl-MS-2* | 3-4mo | F | April 20 2025 | ☑ | ☑ | ☑ | ☑ | ☑ | ☑ | ☑ | X | ☑ |
| *A4 Fl-MS-3* | 3-4mo | F | April 20 2025 | ☑ | ☑ | ☑ | ☑ | ☑ | ☑ | ☑ | X | ☑ |
| | | | | | | | | | | | | |
| *A4 Fl-MS-1* | 4 mo | F | Jun 20 2025 | ☑ | ☑ | ☑ | ☑ | ☑ | ☑ | ☑ | ☑ | X |
| *A4 Fl-MS-2* | 4 mo | F | Jun 20 2025 | ☑ | ☑ | ☑ | ☑ | ☑ | ☑ | ☑ | ☑ | X |
| *CD4CreA4 Fl-MS-1* | 4 mo | F | Jun 20 2025 | ☑ | ☑ | ☑ | ☑ | ☑ | ☑ | ☑ | ☑ | X |
| *CD4CreA4 Fl-MS-2* | 4 mo | F | Jun 20 2025 | ☑ | ☑ | ☑ | ☑ | ☑ | ☑ | ☑ | ☑ | X |
| *A4 Fl-MS-1* | 4 mo | F | Jun 20 2025 | ☑ | ☑ | ☑ | ☑ | ☑ | ☑ | ☑ | X | ☑ |
| *A4 Fl-MS-2* | 4 mo | F | Jun 20 2025 | ☑ | ☑ | ☑ | ☑ | ☑ | ☑ | ☑ | X | ☑ |
| *CD4CreA4 Fl-MS-1* | 4 mo | F | Jun 20 2025 | ☑ | ☑ | ☑ | ☑ | ☑ | ☑ | ☑ | X | ☑ |
| *CD4CreA4 Fl-MS-2* | 4 mo | F | Jun 20 2025 | ☑ | ☑ | ☑ | ☑ | ☑ | ☑ | ☑ | X | ☑ |
| | | | | | | | | | | | | |
| *A4 Fl-MS-1* | 4 mo | F | July 3 2025 | ☑ | ☑ | ☑ | ☑ | ☑ | ☑ | ☑ | ☑ | ☑ |
| *CD4CreA4 Fl-MS-1* | 4 mo | F | July 3 2025 | ☑ | ☑ | ☑ | ☑ | ☑ | ☑ | ☑ | ☑ | ☑ |
| *A4 Fl-MS-1* | 4 mo | F | July 3 2025 | ☑ | ☑ | ☑ | ☑ | ☑ | ☑ | ☑ | X | ☑ |
| *A4 Fl-MS-2* | 4 mo | F | July 3 2025 | ☑ | ☑ | ☑ | ☑ | ☑ | ☑ | ☑ | X | ☑ |
| *A4 Fl-MS-3* | 4 mo | F | July 3 2025 | ☑ | ☑ | ☑ | ☑ | ☑ | ☑ | ☑ | X | ☑ |
| *CD4CreA4 Fl-MS-1* | 4 mo | F | July 3 2025 | ☑ | ☑ | ☑ | ☑ | ☑ | ☑ | ☑ | X | ☑ |
| *CD4CreA4 Fl-MS-2* | 4 mo | F | July 3 2025 | ☑ | ☑ | ☑ | ☑ | ☑ | ☑ | ☑ | X | ☑ |
| *CD4CreA4 Fl-MS-3* | 4 mo | F | July 3 2025 | ☑ | ☑ | ☑ | ☑ | ☑ | ☑ | ☑ | X | ☑ |
| | | | | | | | | | | | | |
| *CD8CreA4 Fl-MS-1* | 4 mo | M | July 14 2025 | ☑ | X | X | X | X | X | X | X | X |
| *CD8CreA4 Fl-MS-2* | 4 mo | M | July 14 2025 | ☑ | X | X | X | X | X | X | X | X |
| *CD8CreA4 Fl-MS-3* | 4 mo | M | July 14 2025 | ☑ | X | X | X | X | X | X | X | X |
| *CD8CreA4 Fl-MS-4* | 4 mo | M | July 14 2025 | ☑ | X | X | X | X | X | X | X | X |
| *CD8CreA4 Fl-MS-5* | 4 mo | M | July 14 2025 | ☑ | X | X | X | X | X | X | X | X |
| *CD8CreA4 Fl-MS-6* | 4 mo | M | July 14 2025 | ☑ | X | X | X | X | X | X | X | X |
| *A4 Fl-MS-1* | 4 mo | M | July 14 2025 | ☑ | X | X | X | X | X | X | X | X |
| *A4 Fl-MS-2* | 4 mo | M | July 14 2025 | ☑ | X | X | X | X | X | X | X | X |
| *A4 Fl-MS-3* | 4 mo | M | July 14 2025 | ☑ | X | X | X | X | X | X | X | X |
| *A4 Fl-MS-4* | 4 mo | M | July 14 2025 | ☑ | X | X | X | X | X | X | X | X |
| | | | | | | | | | | | | |
| *A4 Fl-MS-1* | 4 mo | F | July 17 2025 | ☑ | ☑ | X | X | ☑ | ☑ | ☑ | X | ☑ |
| *A4 Fl-MS-2* | 4 mo | F | July 17 2025 | ☑ | ☑ | X | X | ☑ | ☑ | ☑ | X | ☑ |
| *A4 Fl-MS-3* | 4 mo | F | July 17 2025 | ☑ | ☑ | X | X | ☑ | ☑ | ☑ | X | ☑ |
| *CD8CreA4 Fl-MS-1* | 4 mo | F | July 17 2025 | ☑ | ☑ | X | X | ☑ | ☑ | ☑ | X | ☑ |
| *CD8CreA4 Fl-MS-2* | 4 mo | F | July 17 2025 | ☑ | ☑ | X | X | ☑ | ☑ | ☑ | X | ☑ |
| *CD8CreA4 Fl-MS-3* | 4 mo | F | July 17 2025 | ☑ | ☑ | X | X | ☑ | ☑ | ☑ | X | ☑ |
| *CD8CreA4 Fl-MS-4* | 4 mo | F | July 17 2025 | ☑ | ☑ | X | X | ☑ | ☑ | ☑ | X | ☑ |
| *CD8CreA4 Fl-MS-5* | 4 mo | F | July 17 2025 | ☑ | ☑ | X | X | ☑ | ☑ | ☑ | X | ☑ |

**Supplementary Table 2. Details of mice.** Summary of mouse strains, ages, sexes, tissues collected, and treatment assignments. Checkmarks indicate tissues collected and analyzed; X indicates tissues not collected. PBS and LPS columns indicate treatment group assignment. WT, wild-type; A4 Fl, *Itga4*^fl/fl^; CD4CreA4 Fl, CD4-Cre *Itga4*^fl/fl^ (CD4^Δ*Itga4*^); CD8CreA4 Fl, CD8-Cre *Itga4*^fl/fl^ (CD8^Δ*Itga4*^); MS, mouse; mo, months; PP, Peyer's patches; Sk BM, skull bone marrow; PBS, phosphate-buffered saline; LPS, lipopolysaccharide.

## S3 Table. Antibody reagents for Flow Cytometry Analysis.

| S. No | Reagents | Source | Catalog number |
|---|---|---|---|
| 1. | AF700 anti-human CD3 (Clone SP34-2) | BD Biosciences | Cat#557917 |
| 2. | APC-Cy7 anti-human CD3 (Clone SP34-2) | BD Biosciences | Cat#557757 |
| 3. | BV650 anti-human CD4 (Clone L200) | BD Biosciences | Cat#563737 |
| 4. | BUV805 anti-human CD8 (Clone SK1) | BD Biosciences | Cat#612889 |
| 5. | BUV737 anti-human CD95 (Clone DX2) | BD Biosciences | Cat#564710 |
| 6. | PE/Dazzle 594 anti-human CD28 (Clone CD28.2) | BioLegend | Cat#302942 |
| 7. | APC-Cy7 anti-human CD20 (Clone 2H7) | BioLegend | Cat#302314 |
| 8. | APC-Cy7 anti-human live/dead | invitrogen | Ref#L34976A |
| 9. | PE anti-human CD197 (CCR7) (Clone 3D12) | BD Biosciences | Cat#561008 |
| 10. | BV785 anti-human CD195 (CCR5) (Clone 3A9) | BD Biosciences | Cat#565001 |
| 11. | PE-CF594 Mouse Anti-Human CD196 (CCR6) (Clone 11A9) | BD Biosciences | Cat#564816 |
| 12. | BV711 anti-human CD69 (Clone FN50) | BioLegend | Cat#310944 |
| 13. | FITC anti-human CD49d (Clone HP2/1) | Beckman Coulter | Part no#IM1404U |
| 14. | PE/Dazzle™ 594 anti-human/mouse Integrin β7 (Clone FIB504) | BioLegend | Cat#321226 |
| 15. | PE anti-human Integrin β1 (Clone TS2/16) | BioLegend | Cat#303003 |
| 16. | APC anti-human CD183 (CXCR3) (1C6/CXCR3) | BD Biosciences | Cat#550967 |
| 17. | PECy7 anti-human PD1 (Clone EH12.2H8) | BioLegend | Cat#329918 |
| 18. | FITC anti-human TNF-α (Clone Mab11) | BioLegend | Cat#502906 |
| 19. | PECy7 anti-human IFNγ (Clone B27) | BioLegend | Cat#506518 |
| 20. | PE/Dazzle™ 594 anti-human IL-2 (Clone MO1-17H12) | BioLegend | Cat#500344 |
| 21. | BUV395 anti-mouse Ki67 (Clone B56) | BD Biosciences | Cat#564071 |
| 22. | BUV563 anti-mouse CXCR3 (Clone CXCR3-173) | BD Biosciences | Cat#741438 |
| 23. | BUV661 anti-mouse CD3 (Clone 17A2) | BD Biosciences | Cat#741562 |
| 24. | BUV737 anti-mouse CD44 (Clone IM7) | BD Biosciences | Cat#612799 |
| 25. | BUV805 anti-mouse CD8a (Clone 53-6.7) | BD Biosciences | Cat#612898 |
| 26. | BV605 anti-mouse CD11b (Clone M1/70) | BD Biosciences | Cat#563015 |
| 27. | BV650 anti-mouse CD4 (Clone RM4-5) | BD Biosciences | Cat#563747 |
| 28. | BV711 anti-mouse CD162 (Clone 2PH1) | BD Biosciences | Cat#740746 |
| 29. | BV785 anti-mouse CD45 (Clone 30-F11) | Biolegend | Cat#103149 |
| 30. | PerCP/Cy5.5 anti-mouse CD11a/CD18 (LFA-1) (Clone H155-78) | Biolegend | Cat#141008 |
| 31. | PerCP-eFlour™710 anti-mouse Int β1 CD29 (Clone HMb1-1) | Invitrogen | Cat#46-0291-82 |
| 32. | PE/Dazzle™ 594 anti-mouse CD49d (Clone R 1-2) | BioLegend | Cat#103626 |
| 33. | PE-Cy5 anti-mouse FOXP3 (Clone FJK-16s) | Invitrogen | Cat#15-5773-82 |
| 34. | PE/Cy7 anti-mouse CD279 (PD-1) (Clone RMP1-30) | BioLegend | Cat#109110 |
| 35. | FACS lyse | BD Biosciences | Cat#349202 |
| 36. | FoxP3/ Transcription Factor Staining Buffer set | invitrogen | Cat#00-5523 |
| 37. | Brilliant stain buffer | BD Biosciences | Cat#563794 |

CD: cluster of differentiation; PE: Phycoerythrin; AF: Alexa Fluor; BV: Brilliant violet; BUV: Brilliant ultraviolet; Cy: Cyanine; APC: Allophycocyanin; FITC: Fluorescein Isothiocyanate conjugated

**Supplemental Methods**

**Microscopy** Photomicrographs were systematically acquired from each area using NEUN as anatomical reference. For each PFC slide, a total of 4 photomicrographs were acquired at Layer III along the ventral bank of the prefrontal cortex (PFC), and 4 additional photomicrographs were acquired from the underlying white matter (for a total of 12 PFC gray matter [PFCgm] images and 12 PFC white matter [PFCwm] micrographs). For each hippocampus slide, a total of 4 photomicrographs centered on the pyramidal layer were acquired from the CA1 field, two from the CA3 field, and two from the CA4/hilus field (for a total of 24 hippocampal formation photomicrographs per animal). The experimenter was unable to visualize IBA1 and HLADR channels during area selection to prevent biases.

Photomicrographs were acquired using an upright AxioImager Z2 microscope coupled to an LSM800 confocal head equipped with 2 GaAsP photomultiplier tubes, a 32-channel Airyscan detector, and a laser module capable of generating 405nm, 488nm, 561nm, and 633nm laser lines. All photomicrographs were acquired using a 20X objective (0.8NA), 1024p resolution, 16-bit depth, 2X averaging, 0.76 us pixel time, and the following individual channel settings: DAPI) 0.98AU | 3.5% 405nm laser power | 410-470nm detection wavelength | 500V detector gain | -1,500 detector offset | 0.3 detector digital gain; NEUN) 0.98AU | 5.0% 640nm laser power | 656-700nm detection wavelength | 500V detector gain | 0 detector offset | 1.0 detector digital gain; IBA1) 0.5 AU | 2.0% 488nm laser power | 410-545 detection wavelength | 500V detector gain | -2,560 detector offset | 1.0 detector digital gain; HLADR) 0.51 AU | 5% 561nm laser power | 545-620nm detection wavelength | 750V detector gain | 0 detector offset | 1.5 detector digital gain. All images were saved, unmodified and uncompressed, using Zeiss ZEN 3.8 software in czi format.

**Image Segmentation.** All image processing and quantitative mask generation were performed using a custom Python 3.11 pipeline built around the *scikit-image* (0.22), *numpy* (1.26), *tifffile* (2023.x), and *scipy* (1.11) libraries, following OME-TIFF conversion and voxel size adjustment. The analysis pipeline was executed independently for each scene, producing per-channel 3D binary masks and a per-scene quantitative summary table. Each OME-TIFF file contained four fluorescence channels corresponding to HLA-DR (channel 0), IBA1 (channel 1), DAPI (channel 2), and NEUN (channel 3). All stacks were converted to a canonical C×Z×Y×X format for uniform processing.

To limit computation to the region of optimal tissue focus, each scene was cropped to a *DAPI-based Z-band* determined through a robust coverage analysis. For each Z slice of the DAPI channel, the fraction of above-threshold foreground pixels was computed using an Otsu threshold(66). The per-slice coverage profile was then smoothed with a Gaussian kernel, and a central "plateau" of consistently high coverage was selected. The coverage threshold for inclusion was defined as the median of the central 10% of slices, and the selected region was symmetrically extended by one slice above and below to ensure inclusion of the nuclear boundaries. The resulting cropped interval was applied identically across all channels for downstream segmentation.

The DAPI channel was processed to define nuclear volumes and exclude non-nuclear debris. Each 2D slice was thresholded using the Triangle method(67), which provided robust performance in both sparse and densely stained regions. The resulting binary mask was optionally subjected to a 2D morphological closing (disk radius = 1 pixel) to merge fragmented nuclei. Connected components were then labeled in 3D, and small objects (< 20 $\mu m^3$) were removed. The final DAPI mask thus represented the union of all nuclei within the selected Z-band and was used for downstream normalization and quality control.

IBA1 segmentation was performed using a two-step approach designed to preserve fine microglial processes while maintaining clean somatic boundaries. *Vesselness-enhanced process map(68):*

A 3D Frangi filter (scale range = 1–2 voxels) was applied to enhance tubular structures. Each slice was then thresholded at 20% of its maximum vesselness response. The resulting binary map was lightly dilated (1-pixel radius) to ensure connectivity along fine arbors. *Soma/body mask:* The raw IBA1 intensity was thresholded using Otsu's method and morphologically refined by one iteration of 2D erosion followed by a 2D closing (disk radius = 1 pixel). Small holes (< 15 $\mu m^2$) were filled using a 2D binary fill-holes operation. The two masks were merged by pixelwise maximum operation to generate a single 3D IBA1 mask, which was then pruned of isolated noise components smaller than 20 $\mu m^3$. This fusion approach was empirically optimized to capture both fine processes and cell bodies with minimal background inclusion.

The HLA-DR channel exhibited variable background and uneven illumination, so a multistage adaptive normalization and thresholding strategy was used: *1) Normalization*: Intensities were normalized per slice using percentile stretching between the 3rd and 99.5th percentiles to mitigate inter-slice brightness fluctuations; *2) Background subtraction:* A morphological *rolling ball* filter (69) (radius = 50 pixels) was applied to suppress slow-varying background and emphasize punctate or membranous signal. *3) Adaptive hysteresis thresholding:* High thresholds were computed using the *Yen* method on each slice. The low threshold was set to 90 % of the high threshold. Pixels exceeding the high threshold were immediately classified as foreground, whereas pixels between the two thresholds were included only if connected to a high-threshold region. 4) *Morphological cleanup*: Binary opening (disk radius = 1 pixel) and closing (radius = 1 pixel) were applied in 2D to remove salt-and-pepper noise and bridge small gaps. Connected components smaller than 20 $\mu m^3$ were removed to exclude noise and tissue edge artifacts, respectively. This method was selected over simpler global thresholding to achieve consistent labeling of microglial and vascular HLA-DR across variable immunostaining intensities.

**Microglia Morphological Reconstruction.** Three-dimensional microglial arbors were reconstructed from binary IBA1 masks using a custom Python 3.11 workflow designed to associate each 3D arbor skeleton with its corresponding soma and quantify morphometric and spatial features on a per-cell basis. Soma masks were first refined by removing very small objects and closing small gaps to ensure continuous volumes. Adjacent somata separated by fewer than 2 voxels were merged to correct for over-segmentation. Each connected soma was then uniquely labeled and its centroid and volume computed in physical units ($\mu m^3$).

To isolate microglial processes, a process mask was dilated around each soma and cleared within a thin exclusion shell to prevent double counting at soma boundaries, followed by a light binary closing to connect fragmented branches. A 3D skeletonization (70) pass was then applied, producing a one-voxel-thick representation of each arbor. Any skeleton voxels lying within somata were removed to confine process tracing to the parenchymal compartment. Each skeleton was then assigned to its nearest soma. Skeleton voxels within a small search radius of each soma served as seed points, and a breadth-first propagation (71) was performed through contiguous skeleton pixels to label all connected segments. This ensured that each microglial arbor was traced from its soma outward, preserving topological continuity. Voxels not connected to any soma were ignored. For each soma, the assigned skeleton was used to compute total arbor length (in µm), as well as the number of branch points and endpoints, by convolving the skeleton with a 26-connectivity kernel to estimate local degree.

For quality control, full 3D overlays were also produced as OME-TIFF volumes with RGB channels corresponding to soma and skeleton colorization. Nearest-neighbor distances were used to calculate cell density (cells/$mm^2$), the Clark–Evans $R$ index and $Z$-score for regularity, the Hopkins $H$ statistic for clustering tendency, and Ripley's $L(r) - r$ function across radii from 5–150 µm.

**Statistical Analysis of Microglial Assessments.** All quantitative measurements were summarized at the level of the biological unit (animal). For each animal, values from individual imaging scenes within a given region were averaged to yield a single representative value per metric. Each brain region was treated as an independent variable, with the experimental groups (SIV-, α4, IgG) as the variables of interest. Prior to hypothesis testing, the distribution of each metric was examined for normality and variance homogeneity. Data were evaluated using the Brown–Forsythe test for equality of variances and by assessing skewness ($|Sk| \leq 1$) and kurtosis ($|K| \leq 3$). Metrics satisfying all three criteria were analyzed parametrically; those that failed were further examined after transformation. When appropriate, square-root or $\log(1 + x)$ transformations were applied. If the transformed data met the parametric assumptions, the transformed values were used; otherwise, non-parametric procedures were retained.

Parametric data were analyzed using one-way ANOVA, followed by Tukey's honestly significant difference (HSD) test for pairwise group comparisons. Non-parametric data were analyzed using the Kruskal–Wallis test, followed by Mann–Whitney U tests for pairwise contrasts with Holm's step-down correction for multiple comparisons. Statistical significance was set at $\alpha = 0.05$ for all analyses. Data visualization was performed using a combination of box and violin plots, which display the full distribution of measurements together with the group median and interquartile range. All statistical analyses and visualizations were conducted using custom Python scripts designed for this project.
